# The biosynthesis of *N*-acyalated tryptazolone in *Mycobacterium tuberculosis* and related bacteria

**DOI:** 10.1016/j.jbc.2025.111079

**Published:** 2025-12-20

**Authors:** Julia Kleetz, Jason C. Grigg, Adam A. Hassan, Adriana Ibtisam, Janine N. Copp, Jennifer Lian, Jie Liu, Lindsay D. Eltis

**Affiliations:** 1 Department of Microbiology & Immunology, Life Sciences Institute, The University of British Columbia, Vancouver, British Columbia, Canada; 2 Department of Microbiology & Immunology, Michael Smith Laboratories, The University of British Columbia, Vancouver, British Columbia, Canada

**Keywords:** *Mycobacterium tuberculosis*, lipid, enzyme kinetics, *N*-acyl amino acid, oxazolone, acyltransferase, actinobacteria

## Abstract

The ability of *Mycobacterium tuberculosis* (Mtb) to thrive within its host is due in part to its complex lipid metabolism, aspects of which remain poorly understood. We recently reported the production of *N*-acylated tyrazolones, a class of oxazolones, by the *tyzACB* gene cluster in Mtb. We now report that Mtb also produces *N*-acylated tryptazolones using a second biosynthetic cluster, *trzAS*. TrzA catalyzed the *N*-acylation of l-tryptophan with the highest specificity for C_5:0_-CoA among acyl-CoAs (*k*_cat_/*K*_*m*_ = 2.3 ± 0.3 × 10^3^ M^−1^s^−1^). Similarly, TrzS, comprising a ThiF-like cyclase fused to a flavin-dependent oxidase, catalyzed the ATP-dependent cyclization and O_2_-dependent desaturation of the acylated amino acid to yield an *N*-acylated tryptazolone. Consistent with AlphaFold structural predictions, the D217A and R540A variants of TrzS were deficient in cyclase and desaturase activities, respectively. These variants and substrate-limitation studies established that the order of cyclization and desaturation is obligate, in contrast to the corresponding reactions in tyrazolone biosynthesis. Strains of *Rhodococcus jostii* RHA1 expressing *trzAS* from Mtb, *Mycobacterium smegmatis*, and RHA1, respectively, produced acyl-tryptophan and tryptazolones with different acyl chain lengths, indicating that the homologs have distinct substrate preferences. Using an optimized extraction method, tryptazolones were detected in *M. smegmatis* and RHA1. Finally, the acyl-tryptophan intermediates mainly accumulated in the culture supernatant, whereas tryptazolones were more abundant inside the cells. Elucidating the tryptazolone biosynthesis pathway in Mtb highlights the diversity of oxazolones in mycolic acid–producing bacteria, broadens our understanding of mycobacterial lipid metabolism, and opens exciting avenues for exploring the physiological roles of these small molecules.

Tuberculosis continues to be the most deadly infectious disease globally, causing an estimated 1.25 million deaths and over 10.8 million new cases in 2023 alone (1). The success of *Mycobacterium tuberculosis* (Mtb), the etiological agent of tuberculosis, is due in part to its complex lipid metabolism. This includes the capacity to biosynthesize a rich diversity of lipids, many of which are found in the bacterium’s complex cell envelope and contribute to pathogenesis (2, 3). For example, the cell envelope, which shields Mtb from immune responses and allows it to persist in its host (2, 3), is characterized by an outer mycomembrane that contains mycolic acids, as well as lipids, such as trehalose dimycolates and lipoarabinomannan (3, 4, 5). Trehalose dimycolate helps the *bacillus* evade immune response and causes granuloma formation (6, 7), whereas lipoarabinomannan has various immunomodulatory effects and may contribute to survival and persistence of Mtb in macrophages (8). Related Actinobacteria, such as *Mycobacterium smegmatis* (Msmeg) and *Rhodococcus jostii* RHA1 (hereafter RHA1), share similar lipid profiles, although the lipids generally have shorter chain lengths and less varied modifications (9, 10, 11). Despite its critical importance, annotation of the Mtb lipidome remains a persistent challenge, with 75% to 90% of detected lipid features failing to match mycobacterial database entries (9, 12). Even when using advanced, modern workflows, more features typically remain unidentified than identified (13). This uncharted lipidome highlights Mtb’s unknown biology as well as the potential for discovering new therapeutic targets.

One class of lipids of burgeoning interest is *N*-acyl amino acids and their derivatives. In addition to canonical envelope lipids, bacteria produce lipids and small molecules bearing fatty acyl chains that function predominantly outside membranes. In Mtb, the siderophores mycobactin and carboxymycobactin contain a peptide group, carry fatty acyl moieties, and are exported by dedicated transport systems essential for virulence (14, 15). Many Proteobacteria secrete *N*-acyl homoserine lactones, a class of fatty acyl amides, as quorum signals (16). Secreted *N*-acyl amino acids (*e.g*., stieleriacines) likewise feature fatty-acyl chains and modulate community composition and have antimicrobial properties (17, 18). Bacteria also release lipopeptides, such as surfactin, composed of a β-hydroxy fatty acid linked to a peptide, which act as potent biosurfactants (19). Similarly, some *N*-acyl amino acids that are best known as precursors of glycine and ornithine membrane lipids can also behave as surface-active molecules outside membranes. These are exemplified by the cholesterol-solubilizing and hemolytic *N*-acyl glycine commendamide and by lyso-ornithine lipid, which was identified based on its biosurfactant activity (20, 21). Collectively, these reports underscore that other lipidic small molecules and particularly *N*-acyl amino acids can serve functions beyond being membrane constituents.

Oxazolones are a class of recently discovered lipids produced by the *N*-acylation of tyrosine or phenylalanine followed by cyclization and desaturation to the final compound (22). These lipids were first discovered in a range of Proteobacteria, like *Pseudoalteromonas rubra*, where the *N*-acyltransferase, OxzA, catalyzes the transfer of acyl groups from acyl-CoA to the amino acid. Subsequently, a fusion protein, OxzB, containing a ThiF-like cyclase domain and a flavin-dependent oxidase (FDO) domain, catalyzes the cyclization and desaturation of the *N*-acylated amino acid to form *N*-acyl tyrazolones and phenazolones. This initial report on oxazolone lipids in Proteobacteria led to the discovery of two gene clusters in Mtb that may encode similar biosynthetic pathways (23). More specifically, a sequence similarity network (SSN) of nitroreductases revealed a cluster that includes OxzB and two FDOs from Mtb. Through a combination of *in vivo* and *in vitro* approaches, we demonstrated that one of these FDOs, TyzC (Rv2337c), is involved in the production of C_12:0_-tyrazolones in Mtb, whereas the second FDO, Rv1355c, remained uncharacterized (23). Interestingly, the genomic organization of *rv1355c* is identical to that of *oxzB* and similar to that of *tyzC* (Fig. 1*A*).

**Figure 1:**
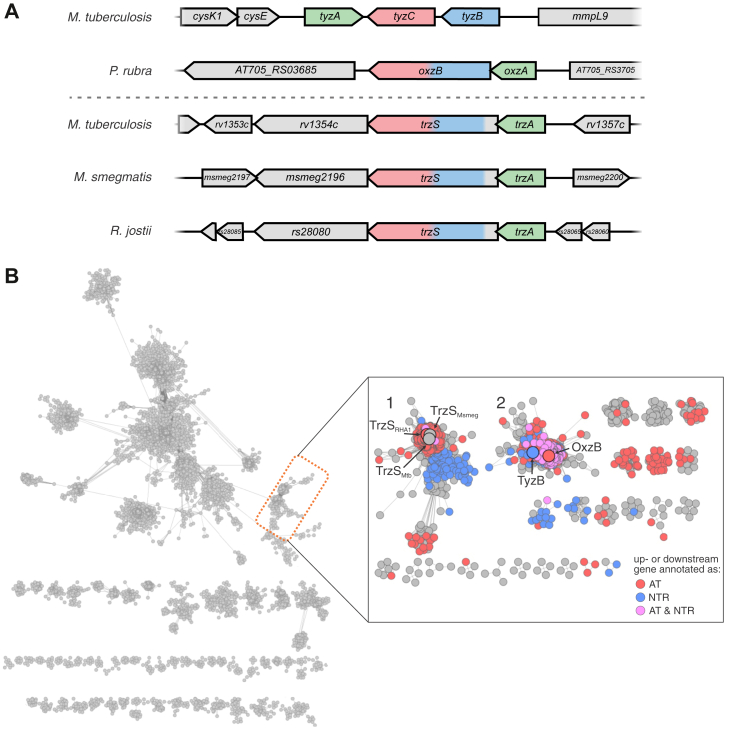
**Bioinformatic analyses of FDO–ThiF fusion proteins.***A*, context of genes encoding or predicted to encode oxazolone biosynthesis enzymes. *B*, a sequence similarity network containing 13,385 nodes representing 130,776 sequences from the ThiF superfamily. Nodes group sequences sharing at least 50% identity, and edges indicate average BLAST values of 1 × 10^−40^. The clusters containing proteins shown or expected to be involved in oxazolone biosynthesis are highlighted and magnified in the inset that contains 2416 sequences at an average blast value of 1 × 10^−65^. Nodes are colored based on the predicted function of the adjacent genes. FDO, flavin-dependent oxidase.

In this study, we combined large-scale SSN analyses, targeted metabolomics, and enzyme kinetics to elucidate the activities of Rv1356c and Rv1355c (hereafter, TrzA_Mtb_ and TrzS_Mtb_, respectively). We investigated the substrate specificities and mechanisms of TrzAS from three mycolic acid–producing bacteria and compared them with those of TyzACB_Mtb_. The demonstration of TrzAS as a tryptazolone biosynthetic pathway broadens the spectrum of known mycobacterial lipids. We discuss the findings with respect to the occurrence of *N*-acylated oxazolones in diverse bacteria and the possible physiological roles of these small molecules.

## Results

### Bioinformatic identification of genes involved in the biosynthesis of oxazolones

We recently established TyzACB as an *N*-acyl tyrazolone biosynthetic pathway in Mtb and identified Rv1355c–Rv1356c as a second potential biosynthetic cluster (23). Based on homology with TyzACB from Mtb (23) and OxzAB from *P. rubra* (22), TrzA (Rv1356c) is a predicted amino acid *N*-acyl transferase, whereas TrzS (Rv1355c) is a predicted fusion of a ThiF-like cyclase and an FDO (Fig. 1*A*). To investigate the function of this putative biosynthetic pathway, we first performed an SSN analysis on the ThiF-like cyclase domain of TrzS. The analysis contained 13,385 nodes representing 130,776 sequences of ThiF-like proteins (Fig. 1*A*). The cluster containing TyzB, OxzB, and TrzS (Fig. 1*B*, *left panel*) was further resolved using a BLAST cutoff of 1 × 10^−65^ (Fig. 1*B*, *right panel*). This SSN contained 2416 sequences and yielded 24 subclusters. Interestingly, the TrzS domain occurred in subcluster 1, distinct from the tyrazolone-producing proteins TyzB and the OxzB domain, which occurred in subcluster 2. To investigate whether more of the proteins in these two subclusters might be involved in oxazolone biosynthesis, we investigated the genomic neighborhood of their encoding genes. Importantly, this analysis relies on computational annotation of gene function. As shown previously, the gene encoding the ThiF protein or domain is either directly adjacent to, or fused to, an FDO-encoding gene and adjacent to an acyltransferase-encoding gene. Coloring the nodes of the subclusters based on the predicted function of their neighboring genes highlights that a majority of nodes represent proteins whose genes are directly adjacent to genes encoding an FDO, an acyltransferase, or both (Fig. 1*B*, *right panel*). This pattern strongly implies that these genes are also involved in oxazolone biosynthesis. In addition to TrzS_Mtb_, subcluster 1 also contained homologs from RHA1 and Msmeg. As in Mtb, these domains occurred as fusions with predicted FDO domains, and their genes occur in putative operons with an upstream *tyzA* homolog (Fig. 1*A*), suggesting that TrzAS synthesizes similar oxazolones in the three mycolic acid–producing strains. Furthermore, this cluster is rich in predicted homologs from mycolic acid–producing Actinomycetota, such as *Mycobacterium*, *Nocardia*, *Gordonia*, and *Rhodococcus* (Fig. S1) (24).

### Substrate preferences of TrzA and TrzS *in vivo*

Based on our bioinformatic analysis, we hypothesized that Mtb, RHA1, and Msmeg possess homologous *trzAS* operons involved in the biosynthesis of similar oxazolones. To test this hypothesis, we turned to an *in vivo* approach. This approach overcame the limited availability of diverse acyl-CoA substrates required for traditional *in vitro* assays and thus facilitates the exploration of substrate preferences of these biosynthetic enzymes. Specifically, we expressed *trzAS* from Mtb, Msmeg, and RHA1 in RHA1, a nonpathogenic, mycolic acid–producing actinobacterium. To eliminate interference from the host’s TrzAS, we constructed an RHA1 Δ*trzAS* double mutant. We then used the pRIME integrative vector to express the different *trzA* homologs under control of a strong constitutive promoter and the pTipQC2 expression plasmid to express the *trzS* homologs under control of an inducible promoter. The recombinant strains were grown in rich medium, and the metabolites were extracted 16 h postinduction from both the cells and the culture supernatant and analyzed using LC–quadrupole time of flight (QTOF). To simplify the analysis, we focused on compounds with fully saturated acyl chains.

**Figure 2:**
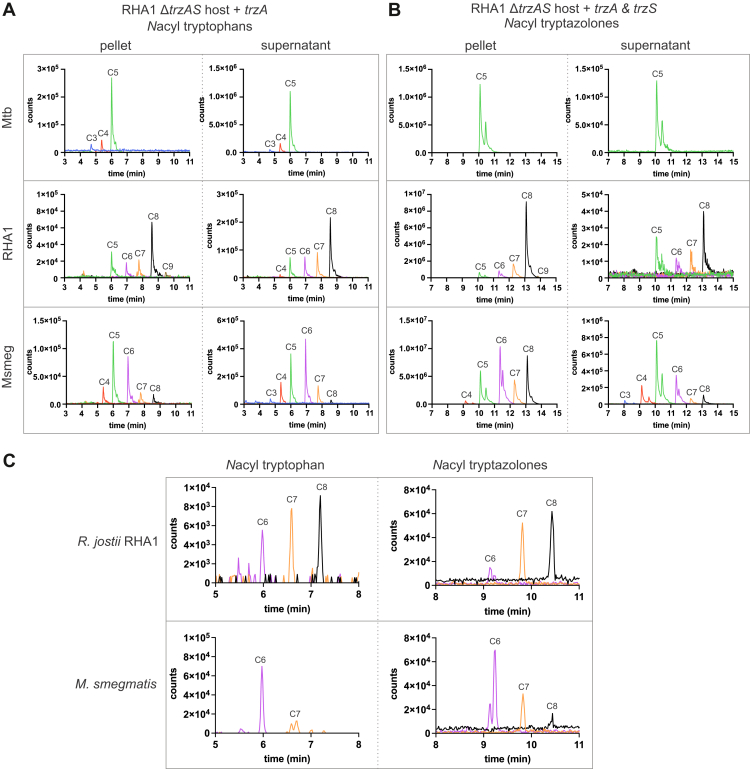
**Acylated****l****-tryptophan and tryptazolones were produced by recombinant and WT RHA1.** The acyltransferases (*A*) and FDO–ThiF fusion proteins (*B*) from Mtb, RHA1, and Msmeg were produced in a *ΔtrzAS* double mutant of RHA1. Metabolites extracted from cell pellets and culture supernatants were analyzed using LC–QTOF. Extracted ion chromatograms (EICs) for detected compounds with the expected *m*/*z* for the [M + H]^+^ ion of acylated l-tryptophans (*left panels*) and tryptazolones (*right panels*) are displayed. For simplicity, only products with fully saturated acyl chains are shown. Detection of products is shown as detector counts against elution time. Chromatograms show representative results from one of three biological replicates. *C*, EICs for compounds corresponding to the expected *m/z* the [M + H]^+^ ion of acylated l-tryptophan (*left panel*) and tryptazolones (*right panel*) detected in WT cells of RHA1 and Msmeg. Chromatograms show representative results from one of at least three biological replicates. FDO, flavin-dependent oxidase; Msmeg, *Mycobacterium smegmatis*; Mtb, *Mycobacterium tuberculosis*; QTOF, quadrupole time of flight; RHA1, *Rhodococcus jostii* RHA1.

When *trzA*_Mtb_ was expressed in the double mutant, three *N*-acylated species were detected: significant amounts of C_5:0_-tryptophan and, in lower amounts, C_3:0_-tryptophan and C_4:0_-tryptophan (Fig. 2*A*). These species were more abundant in the culture supernatant, and no other *N*-acylated amino acids were detected. When both *trzA*_Mtb_ and *trzS*_Mtb_ were expressed, only the C_5:0_-tryptazolone was detected (Fig. 2*B*). In contrast to the acylated tryptophan species, the C_5:0_-tryptazolone was enriched in the cell pellet. As exemplified by analysis of the C_5:0_-acylated species, we detected a small amount of the unsaturated species but none of the cyclized, saturated species (Fig. S2, *A* and *B*). In principle, these intermediates could be produced by the FDO and cyclase domains of the fusion protein, respectively. Importantly, no tyrosine- or phenylalanine-derived species were detected. These results indicate that TrzAS_Mtb_ differs from TyzACB and OxzAB in its specificity for acyl-CoA and amino acid substrates.

Interestingly, TrzA and TrzS from Msmeg and RHA1 yielded slightly different spectra of products when expressed in the Δ*trzAS* double mutant. Thus, a strain harboring TrzA_RHA1_ alone produced a larger variety of acyl-tryptophans, ranging from C_4:0_ to C_9:0_, with C_8:0_ being the most abundant (Fig. 2*A*). A strain harboring TrzA_RHA1_ and TrzS_RHA1_ produced the same range of *N*-acyl tryptazolones (Fig. 2*B*). In contrast, TrzA_Msmeg_ and TrzS_Msmeg_ produced *N*-acylated tryptophan and tryptazolone species with chain lengths ranging from C_3:0_ to C_8:0_ (Fig. 2, *A* and *B*). Notably, while C_8:0_-tryptophan was one of the least abundant acyl-tryptophan species, the corresponding tryptazolone was among the most abundant in cell pellets, suggesting that TrzA_Msmeg_ and TrzS_Msmeg_ have specificities for acylated substrates of differing chain lengths. In all cases, acylated tryptophan species were more abundant in the culture supernatant, whereas tryptazolones were enriched in the cell pellet (Fig. 2, *A* and *B*). Uncyclized, unsaturated species were detected in all strains, whereas cyclized, saturated species were not detected (Fig. S2, *C*–*E*). Moreover, none of the homologs produced acylated oxazolones derived from amino acids other than l-tryptophan.

We next investigated whether the tryptazolones or their precursors could also be detected in the respective WT strains. Based on our experience with the C_12:0_-tyrazolone in Mtb (23), we expected the tryptazolones to be present in small amounts. As discussed later, we also observed that tryptazolones are acid labile (Fig. S3*C*). We therefore compared different lysis and extraction procedures to optimize the detection of oxazolones and their precursors. Tryptazolones were detected in the highest abundance in cell pellets incubated on ice and extracted using butanol–methanol (BUME) (Fig. S3*A*). Interestingly, incubation of the cell pellet at room temperature, sonication, and bead-beating decreased the amount of tryptazolone detected, likely because of the relative instability of these compounds.

Using this optimized extraction method, we detected C_6:0_–C_8:0_ tryptophan and tryptazolones in WT RHA1 pellets from cells grown on rich medium (Figs. 2*C* and S4*A*), closely mimicking the range of species detected in the double mutant expressing the *trzAS*_RHA1_ genes (Fig. 2, *A* and *B*). Investigation of the *N*-acylated tryptophan and tryptazolones in Msmeg cell pellets revealed that the most abundant species were C_6:0_-tryptophan and tryptazolone, followed by lower amounts of C_7:0_ and C_8:0_ acyl chains (Figs. 2*C* and S4*B*). This spectrum differs from the acyl-chain species detected upon heterologous expression of *trzAS*_*Msmeg*_ in RHA1, where C_5:0_-tryptophan, as well as C_5:0_- and C_8:0_-tryptazolone were also quite abundant (Fig. 2, *A* and *B*). This discrepancy demonstrates that while our RHA1 *in vivo* system provides valuable insights into substrate preferences, it is also influenced by the cellular conditions, such as the acyl-CoA pool, and thus needs to be confirmed in the native organism and through *in vitro* experiments. The identity of the most abundant acylated tryptophan and tryptazolone in each bacterium was confirmed by MS/MS (Fig. S5) using acetyl-tryptophan as a standard and the reported fragmentation patterns of tyrazolones as a guide (22, 23).

We had previously investigated the products of TyzACB_Mtb_ produced in WT RHA1 (23). To exclude background activity from the RHA1 TrzAS system, we repeated this experiment using the RHA1 Δ*trzAS* mutant as a host. Overall, the range of acylated tyrosine and tyrazolones produced by TyzACB_Mtb_ in the mutant was very similar to what was produced in WT RHA1. More specifically, C_9:0_- to C_14:0_-tyrazolones were produced in the WT host, whereas C_9:0_- to C_13:0_-tyrazolones were produced in the mutant (Fig. S6, *A* and *B*). In both cases, the most abundant product was C_12:0_-tyrazolone. TyzA_Mtb_ alone produced acylated tyrosines ranging from C_7:0_ to C_14:0_, with C_10:0_ being the most abundant (Fig. S6*A*). Finally, the cyclized intermediate was only detected when the full pathway was expressed and not when only TyzA_Mtb_ and the TyzB_Mtb_ cyclase were present (Fig. S6*C*). Similarly, species with *m*/*z* values of the unsaturated intermediate were only detected at intensities higher than the control expressing only the acyltransferase when all three enzymes were present.

### Heterologous production and *in vitro* activity of TrzA

**Figure 3:**
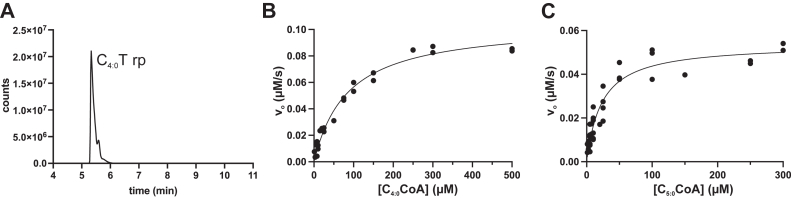
***In vitro* activity of TrzA_Mtb_.***A*, activity of purified enzyme was assessed using l-tryptophan and C_4:0_-CoA. Reactions were quenched with acetonitrile and analyzed by LC–QTOF. Reactions contained 0.5 mM l-tryptophan, 0.1 mM C_4:0_-CoA, and 1 μM of TrzA_Mtb_ (20 mM Tris, 50 mM NaCl, pH 8.0, 21 °C) and were incubated for ∼30 min. EICs for *m/z* values consistent with the [M + H]^+^ ion of C_4:0_-tryptophan product. Detection of products is shown as detector counts against elution time. *B* and *C*, steady-state kinetics of TrzA_Mtb_ using varying concentrations of C_4:0_-CoA (*B*) and C_5:0_-CoA (*C*) determined using a DTNB-based spectrophotometric assay. Reactions contained 0.5 mM l-tryptophan, 1 to 500 μM of acyl-CoA, and 0.5 mM DTNB in 20 mM Mops, pH 7.2, *I* = 100 mM, and were initiated by the addition of 1 μM TrzA_Mtb_. DTNB, 5,5′-dithiobis-(2-nitrobenzoic acid); EIC, extracted ion chromatogram; QTOF, quadrupole time of flight.

Having established that *N*-acyl-tryptazolones are produced *in vivo*, we next sought to dissect the pathway by characterizing the individual enzymes *in vitro*, beginning with the *N*-acyltransferase, TrzA. To validate the function of TrzA, we expressed *trzA*_Mtb_ in *Escherichia coli* as a His_6_-SUMO-tagged protein and purified it using affinity chromatography to >95% apparent homogeneity (Fig. S7*A*). To assess the enzyme’s amino acid acylating activity, we incubated the purified protein with l-tryptophan and butyryl-CoA (C_4:0_-CoA) and analyzed the reaction products using LC–QTOF. We detected a product with the *m*/*z* value of C_4:0_-tryptophan (Fig. 3*A*), confirming that TrzA_Mtb_ functions as an amino acid acyltransferase, similar to OxzA and TyzA.

Next, we assessed the substrate specificity of TrzA_Mtb_ using 5,5′-dithiobis-(2-nitrobenzoic acid) (DTNB, Ellman’s reagent) in a spectrophotometric assay. Briefly, DTNB reacts with free thiol groups that are generated after transfer of an acyl chain from acyl-CoA. The resulting 5-thio-2-nitrobenzoate can be detected at 412 nm. Using a variety of acyl-CoA substrates and tryptophan in 20 mM Mops, pH 7.2, *I* = 100 mM, TrzA_Mtb_ had the highest specific activity for C_4:0_-CoA, followed by C_5:0_-CoA and C_3:0-_CoA (Table 1). The enzyme also used C_6:0_- and C_8:0_-CoAs at lower rates. The DTNB assay was not sensitive enough to measure the turnover of poor substrates. However, LC–QTOF confirmed acylation of tryptophan by long-chain C_12:0_- and C_16:0_-CoA and acylation of phenylalanine with C_4:0_-CoA (Fig. S8). No other amino acids tested were acylated under our conditions. When the concentration of C_4:0_-CoA was varied, TrzA_Mtb_ displayed Michaelis–Menten kinetics with a *K*_*m*_ of 85 ± 10 μM, *k*_cat_ of 10.5 ± 0.4 × 10^−2^ s^−1^, and *k*_cat_/*K*_*m*_ of 1.2 ± 0.2 × 10^3^ M^−1^s^−1^ (Fig. 3*B*). Using C_5:0_-CoA, TrzA_Mtb_ had a *K*_*m*_ of 23 ± 3 μM, a *k*_cat_ of 5.4 ± 0.3 × 10^−2^ s^−1^, and a *k*_cat_/*K*_*m*_ of 2.3 ± 0.3 × 10^3^ M^−1^s^−1^ (Fig. 3*C*). These values are very similar to those reported for TyzA_Mtb_ for C_12:0_-CoA (*e.g.*, *k*_cat_/*K*_*m*_ of 5.9 ± 0.8 × 10^3^ M^−1^s^−1^, (23)). However, TyzA_Mtb_ displayed substrate inhibition kinetics, which we did not observe for TrzA_Mtb_. Finally, the higher substrate specificity of the enzyme for C_5:0_-CoA *versus* C_4:0-_CoA is consistent with C_5:0_-tryptophan being the major product observed *in vivo*.

**Table 1:** Specific activity of TrzA_Mtb_ for various acyl-CoAs^a^

Acyl-CoA	Rate (min^−1^)
C_3:0_	1.21 (0.42)
C_4:0_	6.30 (0.30)
C_5:0_	2.80 (0.10)
C_6:0_	0.74 (0.22)
C_8:0_	0.50 (0.46)

a Activity measured using an assay containing 500 μM l-tryptophan, 250 μM acyl-CoA, 0.5 mM DTNB, and either 1 μM (C_3:0_-, C_4:0_-, and C_5:0_-CoA) or 5 μM (C_6:0-_ and C_8:0_-CoA) TrzA_Mtb_ in 20 mM Mops, pH 7.2 (*I* = 100 mM, 25 °C).

### *In vitro* production of *N*-acylated l-tryptazolone by TrzS

To further characterize the steps of tryptazolone biosynthesis, we attempted to purify TrzS. Despite trying a variety of *E. coli* and RHA1-based expression systems, we were unable to produce TrzS_Mtb_ in sufficient quantity (data not shown). However, we were able to produce TrzS_Msmeg_ as an N-terminally His_10_-tagged protein in *E. coli*. TrzS_Msmeg_, which shares 55.4% amino acid identity with TrzS_Mtb_, was purified using affinity chromatography to >95% apparent homogeneity (Fig. S7*B*). Protein preparations were yellow in color, had absorbance peaks at 355 and 445 nm, and a fluorescence maximum at 530 nm when excited at 440 nm (Fig. S7*F*), consistent with containing FMN (25, 26).

To test whether TrzS_Msmeg_ could utilize the *N*-acylated C_4:0_-tryptophan produced by TrzA_Mtb_ to synthesize tryptazolone, we first examined the reaction using UV–visible spectroscopy since tyrazolones absorb at 360 nm (22, 23). Incubation of TrzA_Mtb_ and TrzS_Msmeg_ with l-tryptophan, butyryl-CoA, ATP, and MgCl_2_ in 20 mM Tris, 50 mM NaCl, pH 8.0, yielded a yellow-colored compound that absorbed strongly at 400 nm (Fig. S3*B*). However, upon quenching with acetic acid and analyzing the products *via* LC–QTOF, the expected tryptazolone was not detected (data not shown). UV–visible spectroscopy of the quenched products further revealed a decrease in the absorbance at 400 nm and an increase near 340 nm (Fig. S3*C*). This prompted us to compare several previously described extraction and quenching methods. We evaluated established protocols such as methanol–chloroform extraction described by Bligh and Dyer (27) and variations thereof, extractions using methyl-*tert*-butyl ether (28) and BUME extraction (29), which we used to detect tyrazolones (23). Our experiment revealed that acids appeared to hydrolyze the oxazolone ring, whereas methanol and ethanol caused alcoholysis, leading to alkylated derivatives (Fig. S3*D*). Importantly, even acetonitrile with 0.1% formic acid, used as a mobile phase during LC–MS, triggered hydrolysis, yielding oxidized C_4:0_-tryptophan as a byproduct. de Rond *et al.* (22) (2021) also observed acid and methanol-based transformation of tyrazolones, indicating that extraction and quenching methods need to be optimized to detect oxazolones. Based on our findings, we quenched *in vitro* reactions with acetonitrile and, as noted above and in our previous study, BUME for cellular metabolite extraction.

**Figure 4:**
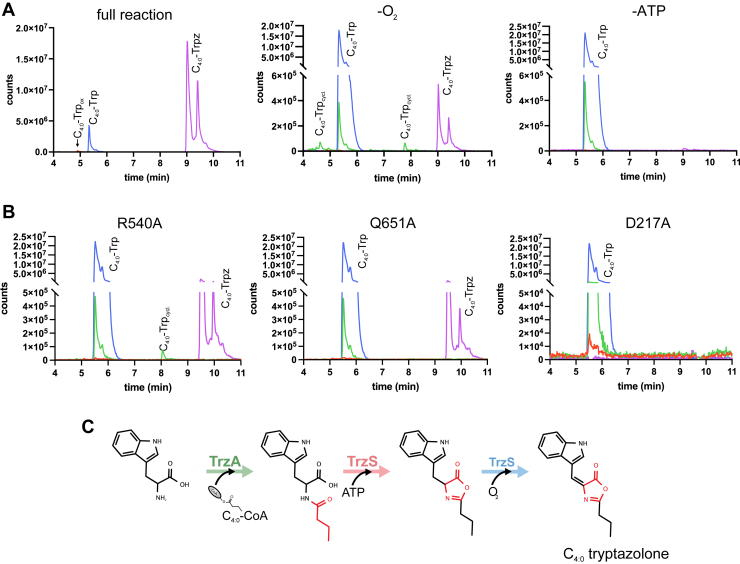
***In vitro* characterization TrzS**_**Msmeg**_**.***A*, EICs for *m/z* values consistent with expected [M + H]^+^ ions of TrzA_Mtb_ and TrzS_Msmeg_ reaction products. Detection of products is shown as detector counts against elution time. Reactions contained 0.5 mM l-tryptophan, 0.1 mM C_4:0_-CoA, 0.5 mM ATP, 1 mM MgCl_2_, and 1 μM of TrzA_Mtb_ and TrzS_Msmeg_ (20 mM Tris, 50 mM NaCl, pH 8.0, 21 °C) and were incubated for ∼30 min. To determine the reaction order, the substrates for the cyclization (ATP) and oxidation (O_2_) reactions were omitted. Reactions were quenched using an equal volume of acetonitrile. Products were analyzed using LC–QTOF. *B*, the activity of TrzS_Msmeg_ variants was tested as described for WT TrzS_Msmeg_. *C*, proposed reaction order of C_4:0_-tryptazolone biosynthesis. EIC, extracted ion chromatogram; QTOF, quadrupole time of flight.

Using acetonitrile instead of acetic acid to quench reactions containing TrzA_Mtb_ and TrzS_Msmeg_, we detected significant amounts of C_4:0_-tryptophan, produced by TrzA_Mtb_, and C_4:0_-tryptazolone (Fig. 4*A*). In addition, small quantities of desaturated C_4:0_-tryptophan were observed (Figs. 4*A* and S9). Importantly, the *N*-acyl tryptazolone was only observed when all components were included in the reaction mixture (Fig. S9). The dependence of the cyclization and desaturation reactions on ATP and O_2_, respectively, provides an opportunity to determine the order of the reaction. When O_2_ was limited in TrzAS reactions, a significant amount of the cyclized, nonoxidized intermediate accumulated. In contrast, only the *N*-acyl tryptophan was observed when ATP was omitted from the reaction (Fig. 4*A*). We confirmed the identity of the detected compounds using MS/MS fragmentation (Fig. S10) and comparison with the fragmentation patterns of acetyl-tryptophan and *N*-acyl tyrazolones and their intermediates (22, 23). Overall, these findings indicate that the cyclization reaction precedes desaturation in the formation of *N*-acyl tryptazolones.

To gain further insight into the structure of TrzS and the reaction order in the fusion protein, we predicted TrzS_Msmeg_’s structure using AlphaFold 3 (30) and tested the predicted catalytic roles of residues in the ATP-dependent cyclase and O_2_-dependent desaturase activities, respectively. The predicted structure of TrzS_Msmeg_ revealed distinct FDO and ThiF domains connected to each other by a poorly defined linker region (Fig. S11). This linker and the N and C termini had lower confidence scores, indicating that these regions may be structurally disordered (Fig. S11*B*). AlphaFold modeling with ATP positioned the substrate in a pocket of the ThiF domain. In the model, Asp217 sits in close proximity to ATP. Based on similarities to the structure of *E. coli* ThiF–ATP complex (PDB ID: 1ZFN, RMSD = 1.84 Å over 118 αCs) and the role of a conserved residue in that enzyme, we hypothesized that Asp217 interacts with the α-phosphate chain of ATP (31). We therefore substituted Asp217 with alanine (D217A) to disrupt ATP binding (Fig. S11*D*). The variant enzyme was purified as described for native TrzS_Msmeg_ (Fig. S7*E*). Preparations were yellow, had an absorbance peak at 445 nm typical of FMN, and fluoresced at 530 nm (Fig. S7*F*). However, this fluorescence was ∼4-fold lower compared to the WT TrzS_Msmeg_, indicating impaired FMN incorporation. In reactions containing TrzA and the D217A variant, only C_4:0_-tryptophan was detected: no *N*-acyl tryptazolone or intermediates were detected (Fig. 4*B*). This is consistent with structural prediction that Asp217 is important for the interaction of the protein with ATP as well as the lack of product observed in the absence of ATP.

Designing mutants to inactivate the desaturase activity was complicated by the lack of FDO substrates for modelling in AlphaFold. However, using Foldseek (32), we identified Acg from Msmeg (Protein Data Bank ID: 2YMV) as the closest structurally characterized FDO homolog (33). Overlaying the AlphaFold model of TrzS_Msmeg_ with Acg (RMSD = 2.26 Å over 178 αCs) suggested a potential binding site for the FMN cofactor of TrzS_Msmeg_ (Fig. S11*C*). We targeted Arg540 and Gln651 as they are in close proximity to the presumed FMN binding site and are likely to contribute to the predicted substrate binding pocket (Fig. S11*D*). Based on this, we constructed and purified the R540A and Q651A variants of TrzS_Msmeg_ (Fig. S7, *C* and *D*). Preparations of these variants had a faint yellow color and minimal absorbance at 445 nm. Moreover, preparations of the Arg540 and Gln651 variants fluoresced at 530 nm, albeit at ∼40- and ∼150-fold lower levels, respectively, compared with the WT protein (Fig. S7*F*). In reactions containing the R540A variant, significantly lesser amounts of tryptazolone were observed than in reactions containing WT TrzS_Msmeg_ (Fig. 4*B*), consistent with low FMN content. In addition, significant amounts of the cyclized, unsaturated intermediate were detected (Fig. 4*B*), consistent with the accumulation of this intermediate under O_2_-limited conditions (Fig. 4*A*). Reactions with the Q651A variant contained reduced amounts of tryptazolone compared with the WT protein (Fig. 4, *A* and *B*). However, in contrast to the R540A variant, no reaction intermediates accumulated. These results confirm that both substitutions impaired catalytic turnover in the FDO domain of the protein.

The obligate order of the TrzS-catalyzed steps contrasts with our findings for TyzC_Mtb_ and TyzB_Mtb_, which indicated that desaturation occurs first (23). However, those results may have been obfuscated by our quenching method, which included acetic acid. We therefore re-examined the order of the TyzC_Mtb_ and TyzB_Mtb_ reactions using acetonitrile as a quencher to minimize hydrolysis (Fig. S3*D*). We conducted endpoint analyses on reactions containing l-Tyr, C_12:0_-CoA, ATP, MgCl_2_, TyzA_Mtb_, and clarified lysate of RHA1 expressing *tyzCB*_Mtb_, as described previously (23). Consistent with our earlier findings, we detected substantial amounts of C_12:0_-Tyr, C_12:0_-tyrazolone, and the desaturated, noncyclized intermediate (Fig. S12*A*). However, in contrast to acetic acid–quenched samples, acetonitrile-quenched samples also contained the cyclized, unsaturated compound (Fig. S12, *A*–*C*). When O_2_ was omitted from the reaction, slightly higher amounts of this compound were detected. When ATP was omitted, slightly higher amounts of the saturated intermediate were detected. These results suggest that in the TyzACB_Mtb_ system, the order of the cyclization and desaturation reactions is not obligate (Fig. S12*D*).

## Discussion

This study establishes that *rv1356c* and *rv1355c* encode TrzA and TrzS, responsible for the biosynthesis of *N*-acyl tryptazolones. TrzAS is the second oxazolone biosynthetic pathway described in Mtb after TyzACB, which produces tyrazolones (23). More specifically, TrzA catalyzes the *N*-acylation of l-tryptophan, whereas TrzS catalyzes the successive cyclization and desaturation of *N*-acyl tryptophan to *N*-acyl tryptazolones, reactions that correspond to those catalyzed by TyzB and TyzC, respectively. The occurrence of *N*-acylated tryptazolones expands the diversity of known oxazolones beyond the previously described tyrazolones and phenazolones (22, 23). Moreover, the SSN analysis suggests that the TrzAS pathway is widely distributed in mycolic acid–producing bacteria (Figs. 1*B* and S1). In fact, more than 50% of the proteins in the subcluster containing TrzS are from Actinomycetota, with mycolic acid–producing genera, such as *Mycobacterium*, *Rhodococcus*, *Gordonia*, and *Nocardia* predominating.

Comparison of the tryptazolones from three different bacterial strains indicates that the acyl moiety is species dependent and that the produced compound appears to be determined by both the cellular pool of available acyl-CoAs and enzyme specificity. Thus, in an RHA1 Δ*trzAS* background, TrzAS_Mtb_ yielded only C_5:0_-tryptazolone, whereas TrzAS_RHA1_ yielded C_5:0_–C_9:0_ species, and TrzAS_Msmeg_ yielded C_3:0_–C_8:0_ tryptazolones (Fig. 2, *A* and *B*). By contrast, no C_5:0_ species were detected in WT Msmeg (Fig. 2*C*) despite C_5:0_-Trp and tryptazolone being major species that accumulated in RHA1-expressing *trzAS*_Msmeg_. Similarly, TrzA_Mtb_ had the highest specificity for C_4:0_-CoA *in vitro*, yet expression in RHA1 produced predominantly C_5:0_-tryptophan and, with TrzS_Mtb_, C_5:0_-tryptazolone, suggesting that intracellular substrate supply influences the product. The different range of products of the acyltransferase *versus* the ThiF–FDO fusion protein is consistent with what was reported for TyzACB_Mtb_ (23). More particularly, TyzCB_Mtb_ acted on a much narrower range of substrates than the acyltransferase TyzA_Mtb_. Overall, these data indicate that the particular acyl-oxazolone produced by a given bacterium depends on the specificities of the respective enzymes as well as the acyl-CoA pool. It will be interesting to determine the range of *N*-acylated oxazolones produced by strains identified in the SSN analysis.

Substrate-limitation and mutagenesis studies established that in TrzS, ThiF-mediated cyclization precedes FDO-catalyzed desaturation. Thus, omission of ATP from the assay or substitution of Asp217, a conserved residue in the ThiF adenylation motif, prevented formation of both cyclized and desaturated products. By contrast, the cyclized, saturated intermediate was produced in assays when oxygen was limited or when the R540A variant of TrzS was used (Fig. 4, *A* and *B*). By contrast, using an optimized acetonitrile quenching method revealed that desaturation and cyclization can proceed in either sequence in the TyzACB_Mtb_ pathway (Fig. S12). The order of these reactions in OxaB has not been reported, and it is unclear whether the obligate order of cyclization and desaturation in TrzS is due to the fused architecture of this enzyme.

A major unresolved question concerns the physiological role of the oxazolones. This question is only heightened by the occurrence of up to two oxazolone biosynthetic pathways in many Actinomycetota. It is possible that the different *N*-acyl oxazolones have different functions, considering that C_12:0_-tyrazolones could occur in membranes, whereas C_5:0_-tryptazolones might not be hydrophobic enough. Interestingly, *N*-acyl tyrosine derivatives secreted by *Stieleria maiorica* Mal15^T^ have antimicrobial activity (17, 18, 34, 35). To date, the described naturally occurring *N*-acyl oxazolones are products of aromatic amino acids. However, oxazolone motifs are not limited to aromatic precursors and have also been described in other bacterial natural products such as methanobactins and the *Haemophilus influenzae* virulence factor oxazolin, which can be derived from cysteine residues (36, 37). The *N*-acyl oxazolones are also structurally similar to cell–cell signaling molecules, such as acyl homoserine lactones (16, 38, 39, 40), hydroxyquinolones (41), and cyclic dipeptides (42). Consistent with this possible role, the *trzAS* locus is adjacent to genes predicted to be involved in cyclic di-GMP turnover (*rv1354c* and *rv1357c* in Mtb). However, we have so far not been able to detect the tryptazolones in Mtb cell pellets or culture supernatants despite detecting them in the closely related species Msmeg and RHA1 (Fig. 2*C*). Similarly, although subinhibitory antibiotic concentrations induced oxazolone production in several proteobacteria (22), neither antibiotics nor oxidative stress increased tyrazolone production or induced tryptazolone production in Mtb (23). This inducibility in Proteobacteria, together with prior genetic links between the Mtb *tyz* locus and oxidative-stress response (43) and macrophage survival (44), is consistent with the previously proposed hypothesis that oxazolones contribute in some manner to stress-associated physiology. While the *trzAS* genes are predicted to be nonessential for both growth and pathogenesis (45, 46, 47), *trzS* expression was positively associated with drug resistance (48), and *trzA* was more highly expressed in drug-resistant isolates (49). Together, these observations suggest that oxazolones may link environmental cues to stress adaptation.

Overall, by demonstrating that TrzAS specifies the biosynthesis of *N*-acylated tryptazolone, this study expands the known chemical diversity of mycobacterial oxazolones and broadens our understanding of mycobacterial lipid metabolism. Moreover, the tryptazolones are predicted to be widely distributed. The improved extraction methods described herein, the host-dependent chain-length profile, and the intracellular localization of the tryptazolones provide a conceptual framework for probing the physiological role of these fascinating lipids.

## Experimental procedures

### Bioinformatic analyses

SSN was performed as previously described (23, 50, 51). Briefly, protein sequences classified under the ThiF superfamily were retrieved using the InterPro (52) category IPR000594 (ThiF-type NAD/FAD binding fold). Representative UniProt50 sequences were used to create SSNs using EFI–EST (53, 54), applying an average BLAST score of 1 × 10^−40^ or 1 × 10^−65^. The genomic context of genes was visualized based on results from blastP (55) and BioCyc (56), with *oxzAB* from *P. rubra* (22) and *tyzACB*_Mtb_ (23) serving as template sequences.

### Bacterial strains and growth media

Strains used in this study are listed in Table S2. Briefly, *E. coli* DH5α was used to propagate DNA. TrzS_Msmeg_ and TrzA_Mtb_ were overproduced in *E. coli* BL21(DE3) and *Escherichia cloni* (Lucigen), respectively. *E. coli* S17.1 served as the donor strain for conjugation with *R. jostii* RHA1. *R. jostii* RHA1 was used to detect oxazolones and as a host to produce TrzS_Msmeg_, TrzS_Mtb_, and TrzS_RHA1_. Msmeg mc^2^-155 was used to detect oxazolones. Strains were routinely grown in LB broth or on LB agar at 30 °C (RHA1) or 37 °C (*Msmeg* and *E. coli*). Cultures were supplemented with appropriate antibiotics at the following concentrations: 50 μg/ml kanamycin (pET28a, pK19mobsacB), 30 μg/ml chloramphenicol (pTip), and 30 μg/ml apramycin (pRIME).

### DNA manipulation

Plasmids used and created in this study are listed in Table S1. Oligonucleotides used are listed in Table S2. DNA was propagated, purified, and manipulated using standard protocols (57). Plasmids were created using Gibson Assembly. For the construction of pET28a-TrzS_Msmeg_, the *trzS* gene was amplified from Msmeg mc^2^-155 genomic DNA and cloned into pET28a-10xHIS-tobacco etch virus using the NdeI and XhoI restriction sites. For the construction of pET28a encoding TrzS point mutants, site-directed mutagenesis was performed by Gibson Assembly using NEB Gibson Assembly Master Mix according to the manufacturer’s instructions. The *trzS* gene was amplified in two overlapping fragments using primers introducing the desired nucleotide substitutions. The fragments and NdeI and XhoI linearized vector were assembled as described above. For the overproduction of TrzA_Mtb_ in *E. cloni*, the gene was amplified from Mtb H37rv genomic DNA and cloned into pExpresso using the NdeI and BamHI restriction sites. For pTipQC2 plasmids expressing *trzS*, the genes were amplified from the respective genomic DNA and cloned into pTipQC2 that was previously digested with NdeI and XhoI. Integrative pRIME plasmids expressing *trzA* under the control of the M6 promoter were constructed by cloning the genes, amplified from the respective genomic DNA, into pRIME digested with NdeI and SpeI restriction enzymes.

To create marker-free gene deletions in RHA1, flanking regions of *trzS* contained the first 99 bp of the gene as well as 900 bp upstream of the start codon, and the last 99 bp of the gene with an additional 900 bp downstream of the stop codon (Fig. S13*A*). The resulting plasmid was transformed into RHA1 by biparental mating using *E. coli* S17.1 as donor (58, 59). Successful first crossover events were selected based on kanamycin resistance and sucrose sensitivity. Colonies showing this phenotype were subsequently grown on LB agar containing 10% sucrose to select for the second homologous crossover event. Successful gene deletions were confirmed by colony PCR (Fig. S13*B*) and sequencing of the genomic region. The *trzA* gene was deleted using the RHA1 WT as a genetic background. The Δ*trzA* mutant was then used to create the RHA1 Δ*trzA* Δ*trzS* double deletion strain.

### Metabolite extractions from native hosts

For the extraction of *N*-acylated tryptophan and tryptazolones in RHA1 or Msmeg, the bacteria were cultivated in 100 ml (Msmeg) or 10 ml (RHA1) LB and grown until the stationary phase. Cell pellets were harvested by centrifugation and washed once with water. Metabolites were extracted using a modified BUME method (1:1 [v/v] 1-BUME with 5 mM ammonium formate, pH 6.5) as described before (23, 29) and analyzed by LC–QTOF as described below.

### Metabolite extractions from RHA1 producing Trz and tyz proteins

Metabolites were extracted from RHA1 as previously described (23). Briefly, 75 ml of LB broth was inoculated with cells to an absorbance of 0.1 at 600 nm, incubated at 30 °C until an absorbance of ∼0.6 at 600 nm was reached, and protein production was subsequently induced by the addition of 2 μg/ml thiostreptone. After an additional 16 h at 30 °C, cultures were harvested by centrifugation (SLK-3000, 4000 rpm, 15 min, 4 °C). Cell pellets were washed once in water, and culture supernatants were lyophilized. Metabolites from cell pellets and lyophilized supernatants were extracted using a modified BUME method (1:1 [v/v] 1-BUME with 5 mM ammonium formate, pH 6.5) (23, 29) and analyzed by LC–QTOF as described later.

### Protein purification

To purify TrzS_Msmeg_ and mutated variants, *E. coli* BL21 transformed with the respective pET28a plasmid encoding the gene was cultivated overnight in 4 ml LB supplemented with kanamycin. A total of 4 × 1 l of fresh LB with kanamycin was inoculated with 0.5 ml each of the overnight culture and grown at 30 °C until an absorbance of 0.6 at 600 nm was reached. Cultures were then incubated on ice for 1 h, after which 1 mM of IPTG was added to induce protein production and the cultures were cultivated for a further 18 h at 30 °C. Cells were harvested by centrifugation (SLK-3000, 4000 rpm, 15 min, 4 °C), and pellets were used directly or stored at −80 °C until use. For purification, cell pellets were suspended in 30 ml buffer A (0.1 M Tris, pH 8.0, 0.2 M NaCl, 0.5 mM Tris(2-carboxyethyl)phosphine) containing 10 mM imidazole and 0.125 mg/ml DNase I. Cells were lysed by passing through an Emulsiflex C5 homogenizer (Avestin) three times in succession. The cell debris was pelleted by centrifugation (SS-34, 16,000 rpm, 45 min, 4 °C), and the supernatant applied to a nickel–nitrilotriacetic acid (Qiagen) column. The recombinant, His-tagged protein bound to the nickel resin was washed by 10 column volumes of buffer A containing 50 mM imidazole and five column volumes of buffer A containing 80 mM imidazole. The bound protein was subsequently eluted using buffer A containing 500 mM imidazole. Eluted fractions with a yellow color (because of the FMN cofactor of TrzS) were pooled, dialyzed into 20 mM Tris, pH 8.0, 0.2 M NaCl, 0.5 mM Tris(2-carboxyethyl)phosphine, and concentrated to ∼10 mg/ml using an Amicon 30 molecular weight cutoff spin column. The concentrated protein was flash frozen in liquid nitrogen and stored at −80 °C. UV–visible spectra of purified proteins were determined using a Cary 60 spectrophotometer. Fluorescence emission spectra were recorded using a 96-well plate, a TECAN Spark microplate reader, and an excitation wavelength of 440 nm.

Purified TrzA_Mtb_ was prepared as described for TyzA (23), except that the expression of *trzA* was induced using 0.1% (w/v) rhamnose and cultures were subsequently incubated at 20 °C for 18 h. Purified TyzA and lysate of cells expressing TyzBC were prepared as described before (23).

### *In vitro* activity assays

*In vitro* reactions using purified TrzA_Mtb_ and TrzS_Msmeg_ contained 0.5 mM l-tryptophan, 0.1 mM C_4:0_-CoA, and, in the case of TrzS_Msmeg,_ 0.5 mM ATP, 1 mM MgCl_2_, as well as 1 μM of TrzA_Mtb_ and TrzS_Msmeg_ in 20 mM Tris, 50 mM NaCl, pH 8.0. Reactions were incubated at 21 °C for 30 min. To determine the reaction order and required substrates, each reaction component was omitted from the reaction. The effect of oxygen was tested by performing the reaction in a glovebox with a N_2_ atmosphere. Reactions of TyzA alone and together with TyzC–TyzB were performed as described previously (23). Reactions were routinely quenched with equal volumes of 100% acetonitrile, after which samples were centrifuged (20 min, 4 °C, 13,000 rpm) and metabolites analyzed by LC–QTOF as described later.

### Steady-state kinetics for TrzA

Steady-state kinetics were determined using DTNB (Ellman’s reagent) in a photometric assay as described previously (60). As DTNB reacts with the liberated thiol group of CoA upon transfer of the acyl-chain, it forms 5-thio-2-nitrobenzoic acid, which can be detected at a wavelength of 412 nm and quantified using its extinction coefficient (ε = 14,100 M^−1^ cm^−1^). Reactions contained 0.5 mM l-tryptophan, 1 to 500 μM of acyl-CoA and 0.5 mM DTNB in 20 mM Mops, pH 7.2, *I* = 100 mM, and were initiated by the addition of 1 μM TrzA_Mtb_. The reaction was monitored using a Cary 60 spectrophotometer equipped with a thermostatted cuvette holder at 25 °C for 3 min. We verified that none of the other components (DTNB, l-tryptophan, and TrzA_Mtb_) were rate limiting by testing different concentrations. Steady-state kinetic parameters were determined by fitting a model for Michaelis–Menten kinetics using GraphPad Prism. Specific activity of TrzA_Mtb_ was determined as described above, but with a fixed concentration of 250 μM acyl-CoA.

### LC–QTOF

LC–MS analysis was performed on an Agilent 1290 Infinity II UHPLC coupled to an Agilent 6546 Q-TOF with a dual AJS electrospray ionization source, following Grigg *et al.* (23). Samples (2 μl) were injected onto a Zorbax Eclipse Plus C18 column (100 mm × 2.1 mm, 1.8 μm) and separated at 0.45 ml min^−1^ using a 20-min linear gradient from 5% to 100% acetonitrile containing 0.1% formic acid. Metabolite extracts from WT RHA1 and Msmeg pellets were analyzed using the same workflow but using a shortened method on a Zorbax Eclipse Plus C18 RRHD column (50 mm × 2.1 mm, 1.8 μm) at 0.25 ml min^−1^ with a 12-min 5% to 100% acetonitrile (0.1% formic acid) gradient. Generally, data were acquired in positive ionization mode using the following settings: capillary 3500 V, nozzle 500 V, drying gas 300 °C (10 l min^−1^), sheath gas 350 °C (12 l min^−1^), nebulizer 45 psi, and fragmentor 100 V. MS/MS was collected on selected ions at 10, 20, and 40 V collision energies. Data were acquired and analyzed in MassHunter Workstation v10 (Agilent Technologies).

## Data availability

All data are contained within the article and supporting information.

## Supporting information

This article contains supporting information (23, 30, 58, 59, 61, 62, 63, 64).

## Conflict of interest

The authors declare that they have no conflicts of interest with the contents of this article.
