# Supplementary material for: The biosynthesis of N-acyalated tryptazolone in Mycobacterium tuberculosis and related bacteria
Source: J Biol Chem. 2025 Dec 20;302(2):111079. doi: 10.1016/j.jbc.2025.111079 (PMC12856293; doi:10.1016/j.jbc.2025.111079)
Supplement: Supporting Tables S1 and S2, and Figures S1–S13 [file mmc1.docx]

# Supporting information

**Table S****1. Strains and Plasmids used in this study.**

| Strain | Description | Source |
| --- | --- | --- |
| *E. coli* DH5α | DNA propagation | - |
| *E. cloni* | Protein production | Lucigen |
| *E. coli* BL21 (DE3) | Protein production | (1) |
| *E. coli S17.1* | Conjugation of plasmids into RHA1 | (2) |
| *R. jostii* RHA1 | Wild-type | (3) |
| *R. jostii* RHA1 Δ*trzS* | Marker-less deletion of *trzS* | This study |
| *R. jostii* RHA1 Δ*trzA* Δ*trzS* | Marker-less deletion of Δ*trzA* and Δ*trzS* | This study |
| *M. smegmatis mc^2^-155* | Wild-type, detection of oxazolones | NCBI 246196 |
| **Plasmids** |  |  |
| pET28a-10xHIS-TEV | Protein production, kanamycin^R^ | Novagen, Natalie Strynadka (UBC) |
| pET28a-trzS_Msmeg_ | pET28a harboring *trzS*_Msmeg_ | This study |
| pET28a-trzS_Msmeg_D217A | pET28a harboring *trzS*_Msmeg_ with D217A point mutation | This study |
| pET28a-trzS_Msmeg_R540A | pET28a harboring *trzS*_Msmeg_ with R540A point mutation | This study |
| pET28a-trzS_Msmeg_Q651A | pET28a harboring *trzS*_Msmeg_ with Q651A | This study |
| pExpresso | Protein production, kanamycin^R^ | Lucigen |
| pExpresso-SUMO-tyzA_Mtb_ | pExpresso harboring *tyzA*_Mtb_ | (4) |
| pExpresso-SUMO-trzA_Mtb_ | pExpresso harboring *trzA*_Mtb_ | This study |
| pTipQC2 | Protein production, chloramphenicol^R^ | (5) |
| pTip trzS_Msmeg_ | pTipQC2 harboring *trzS*_Msmeg_ | This study |
| pTip trzS_Mtb_ | pTipQC2 harboring *trzS*_Mtb_ | This study |
| pTip trzS_RHA1_ | pTipQC2 harboring *trzS*_RHA1_ | This study |
| pTip tyzBC_Mtb_ | pTipQC2 harboring *tyzBC*_Mtb_ | (4) |
| pRIME M6 | Insertion vector, apramycin^R^, M6 promoter | (6) |
| pRIME trzA_Msmeg_ | pRIME harboring *trzA*_Msmeg_ | This study |
| pRIME trzA_Mtb_ | pRIME harboring *trzA*_Mtb_ | This study |
| pRIME trzA_RHA1_ | pRIME harboring *trzA*_RHA1_ | This study |
| pRIME-tyzA_Mtb_ | pRIME harboring *tyzA*_Mtb_ | (4) |
| pK18mobsacB | Suicide vector for creation of deletion mutants | (2, 7) |
| pK18mobsacB trzS_RHA1_ | pK18mobsacB harboring upstream and downstream flanking regions of *trzS*_RHA1_ | This study |
| pK18mobsacB trzA_RHA1_ | pK18mobsacB harboring upstream and downstream flanking regions of *trzA*_RHA1_ | This study |

**Table S****2. Oligonucleotides used in this study.**

| Name | Sequence | Restriction site |
| --- | --- | --- |
| pET-TrzSMsmeg-F | AACCTGTATTTTCAGGGCCATATGATCTCAGACGACCACCGATTC | NdeI |
| pET-TrzSMsmeg-R | AGTGGTGGTGGTGGTGGTGCTCGAGTTACGACGTCCTGACCCC | XhoI |
| pEX-TrzAMtb-F | CTATAAGAAGGAGATATACATATGTGTTAATTGCCGGATACCTAAC | NdeI |
| pEX-TrzAMtb-R | GTCGACGGAGCTCGAATTCGGATCCTCACTGTTCGGCTCCAGC | BamHI |
| pTip_TrzSMsmeg-F | GGTGGTCGTCTGAGATCATATGTATATCTCCTTCTTAAAG | NdeI |
| pTip_TrzSMsmeg-R | AGTGATGGTGATGGTGATGCTCGAGTTACGACGTCCTGACCCC | XhoI |
| pTip_TrzSMtb-F | CTTTAAGAAGGAGATATACATATGCTATCTGTGCGGCTCTGG | NdeI |
| pTip_TrzSMtb-R | AGTGATGGTGATGGTGATGCTCGAGTATGACCATCCCACACGAG | XhoI |
| pTip_TrzSRHA1-F | CTTTAAGAAGGAGATATACATATGAACGAATCCCGCGGTGAC | NdeI |
| pTip_TrzSRHA1-R | AGTGATGGTGATGGTGATGCTCGAGTTACGATATCCGCCTGCG | XhoI |
| pRIME_TrzAMsmeg-F | CTTTAAGAAGGAGATATACAATGGACACCGACATGACCCTTG | - |
| pRIME_TrzAMsmeg-R | CACGGGTGCCGGTGGGTCGATCATTGCCCCGCCGCAGT | - |
| pRIME_TrzAMtb-F | CTTTAAGAAGGAGATATACATATGATGGTGTTAATTGCCGGATACCT | NdeI |
| pRIME_TrzAMtb-R | CCGCTGGAGCCGAACAGTGAACTAGTTCACGAGACGCTCCGCATC | SpeI |
| pRIME_TrzARHA1-F | CTTTAAGAAGGAGATATACATATGATGTCTGTTGAACAGCTGACGAATC | NdeI |
| pRIME_TrzARHA1-R | CACGGGTGCCGGTGGGTCGACTAGTTCACGAGACGCTCCGCATC | SpeI |
| trzS-upstream-F | ACAGCTATGACATGATTACGAATTCTCCGCAGATAGACGTCTGG | EcoRI |
| trzS-upstream-R | CGAGTTCCGCGTACGCCCACACCGGAGT | - |
| trzS-downstream-F | GTGGGCGTACGCGGAACTCGAACAAGTCTC | - |
| trzS-downstream-R | TAAAACGACGGCCAGTGCCAAGCTTTCGTCGGTCTGGCTGGTC | BamHI |
| trzA-upstream-F | ACAGCTATGACATGATTACGAATTCAACTCGTATTACACGCAAATCGTGG | EcoRI |
| trzA-upstream-R | ACAGTTGGGCACACTCCGACCGCGGATC | - |
| trzA-downstream-F | GTCGGAGTGTGCCCAACTGTTACTCACGACGTCCCGAC | - |
| trzA-downstream-R | CCTGCAGGTCGACTCTAGAGGATCCTGGGCGAGCAGGGCGGCG | HindIII |
| pET-TrzSMsmeg-D217A-1-F | AACCTGTATTTTCAGGGCCATATGATCTCAGACGACCACC | NdeI |
| pET-TrzSMsmeg-D217A-1-R | GAGAGGCGCATTCCTCGACGACGACGTC | - |
| pET-TrzSMsmeg-D217A-2-F | CGTCGAGGAATGCGCCTCTCTCGACGCG | - |
| pET-TrzSMsmeg-D217A-2-R | AGTGGTGGTGGTGGTGGTGCTCGAGTTACGACGTCCTGACCCCC | XhoI |
| pET-TrzSMsmeg-Q651A-1-F | See pET-TrzSMsmeg-D217A-1-F | - |
| pET-TrzSMsmeg- Q651A-1-R | CCGGCGCCACAGAGACACCGTGCTGCTG | - |
| pET-TrzSMsmeg- Q651A-2-F | CGGTGTCTCTGTGGCGCCGGTGTCGCCG | - |
| pET-TrzSMsmeg- Q651A-2-R | AGTGGTGGTGGTGGTGGTGCTCGAGTTACGACGTCCTGACCCCCTGGAG | XhoI |
| pET-TrzSMsmeg-R540A-1-F | AAAACCTGTATTTTCAGGGCCATATGATCTCAGACGACCACCG | NdeI |
| pET-TrzSMsmeg- R540A-1-R | AATACGCGATGCGATCGG TTCGGCGAG | - |
| pET-TrzSMsmeg- R540A-2-F | AACCGATCGCATCGCGTATTTGACGCCC | - |
| pET-TrzSMsmeg- R540A-2-R | See pET-TrzSMsmeg-D217A-2-R | - |


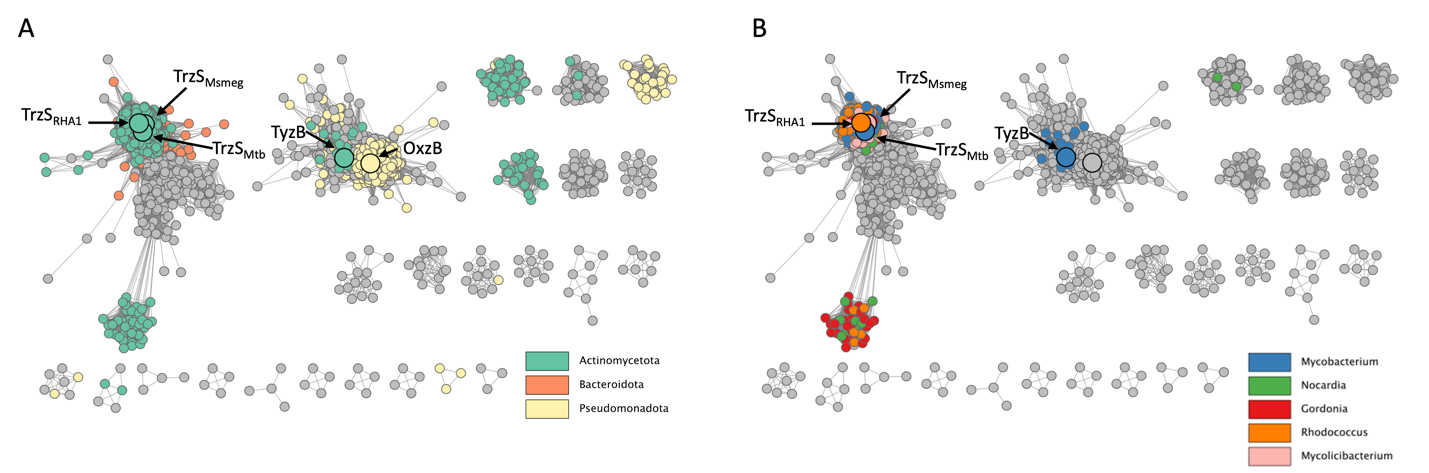


**Figure S****1. Sequence-similarity networks (SSNs) of ThiF subclusters containing oxazolone biosynthesis proteins.** The SSNs were created as described for Fig. 1B. Nodes are colored based on the bacterial phylum (**A**) or genus (**B**) as annotated in the UniProt database.


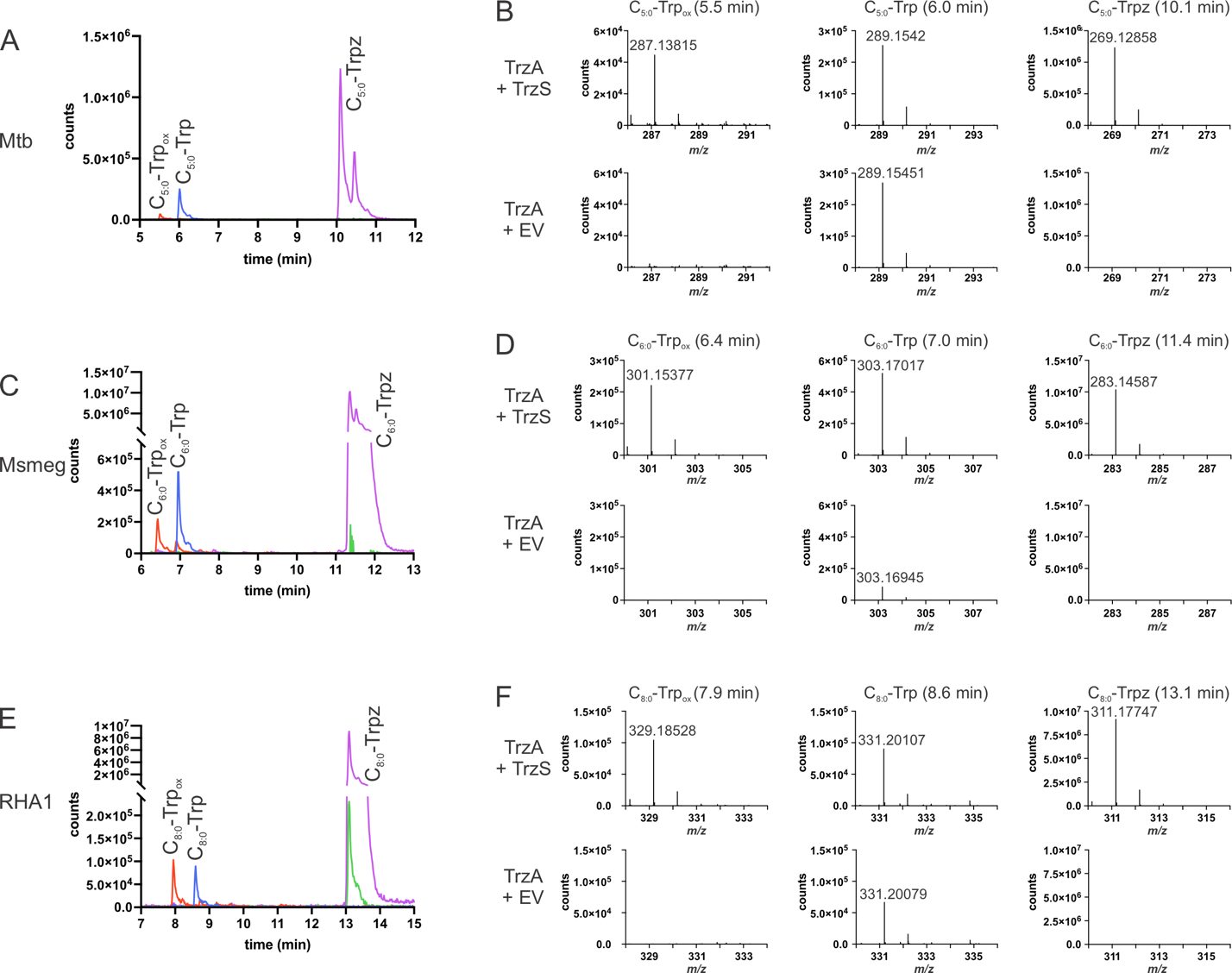


**Figure S****2. Production of the most prominent tryptazolone and respective intermediates after heterologous production of TrzA and TrzS in RHA1**. EICs (**A**/**C**/**E**) and respective mass spectra (**B**/**D**/**F**) for the [M+H]^+^ ion of detected tryptazolones and reaction intermediates. Detection of products is shown as detector counts against elution time (EICs) or respective *m/z* values and indicated elution times. Theoretical *m*/*z* values*:* C_5:0_-Trp, 289.15521; C_5:0_-Trp_ox_, 287.13956; C_5:0_-Trpz, 269.129; C_6:0_-Trp, 303.17087; C_6:0_-Trp_ox_, 301.15521; C_6:0_-Trpz, 283.14465; C_8:0_-Trp, 331.20217; C_8:0_-Trp_ox_, 329.18652; and C_8:0_-Trpz, 311.17595. EV, empty vector.


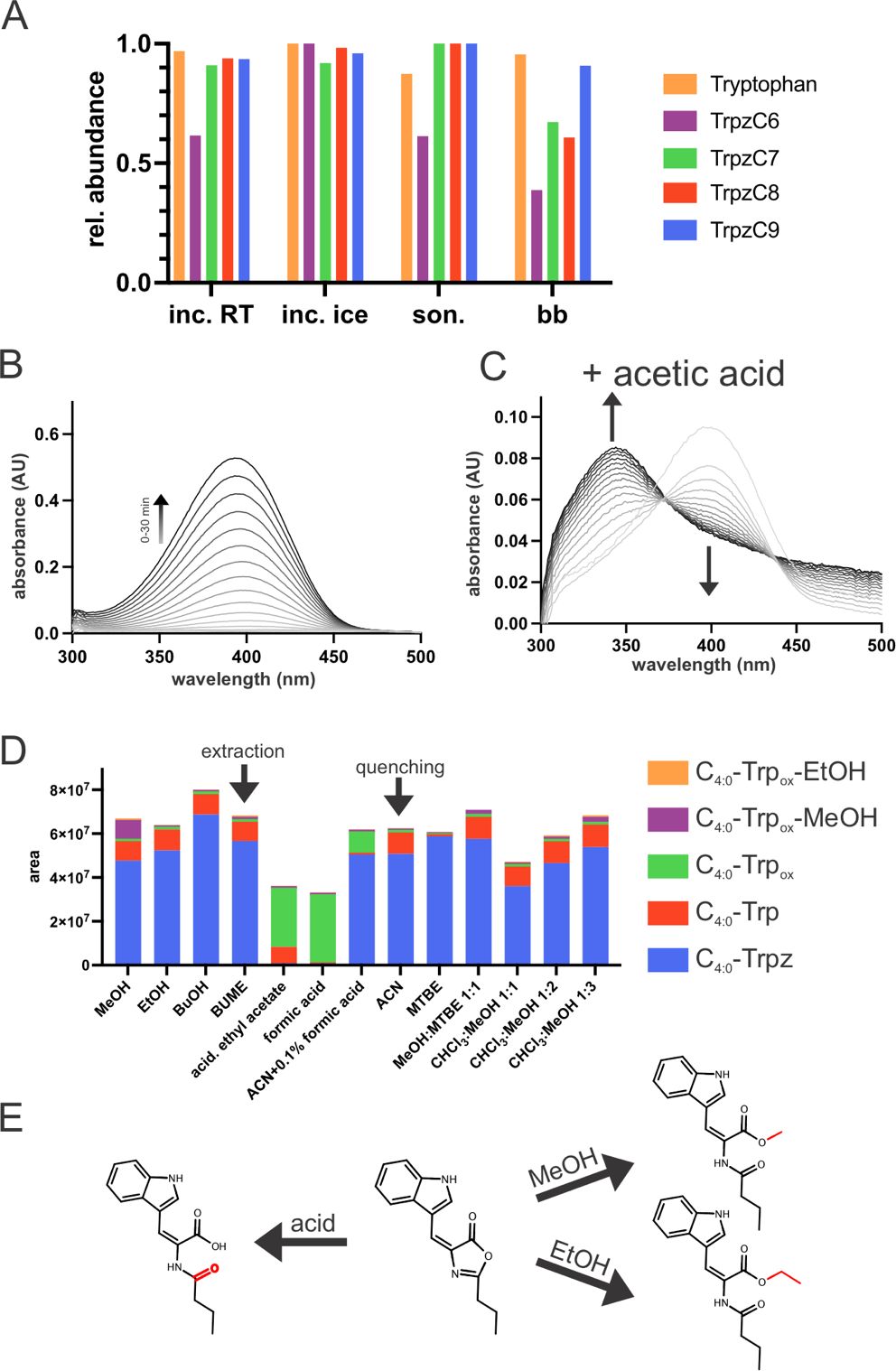


**Figure S****3. Optimization of extraction methods for tryptazolones and intermediates**. **A**) Comparison of relative abundances of compounds detected in RHA1 after butanol-methanol extraction using different lysis methods. Methods were: inc. RT, incubation at room temperature; inc. ice, incubation on ice; son, incubation in ice-cooled sonication water bath; bb, bead beating (see Experimental Procedures for more details). **B**) Monitoring of *in vitro* reaction of TrzAS, performed as described in Experimental Procedures. Spectra recorded every 120 s. **C**) Effect of the addition of acetic acid to 10% (v/v) to the sample in (B) on the absorbance peak at 400 nm. **D**) LC-QTOF analysis of products from *in vitro* reactions using TrzA_Mtb_ and TrzS_Msmeg_ as described before (see Fig. **2**). Methods subsequently used for extraction of compounds from living cells and quenching of *in vitro* reactions are indicated by an arrow. Compounds were quantified as area under the curve from EICs of the expected *m/z* for the [M+H]^+^ ion of all products. **E**) Overview of presumed chemical modification of alcohols methanol (MeOH), ethanol (EtOH), and acid on acylated tryptazolones.


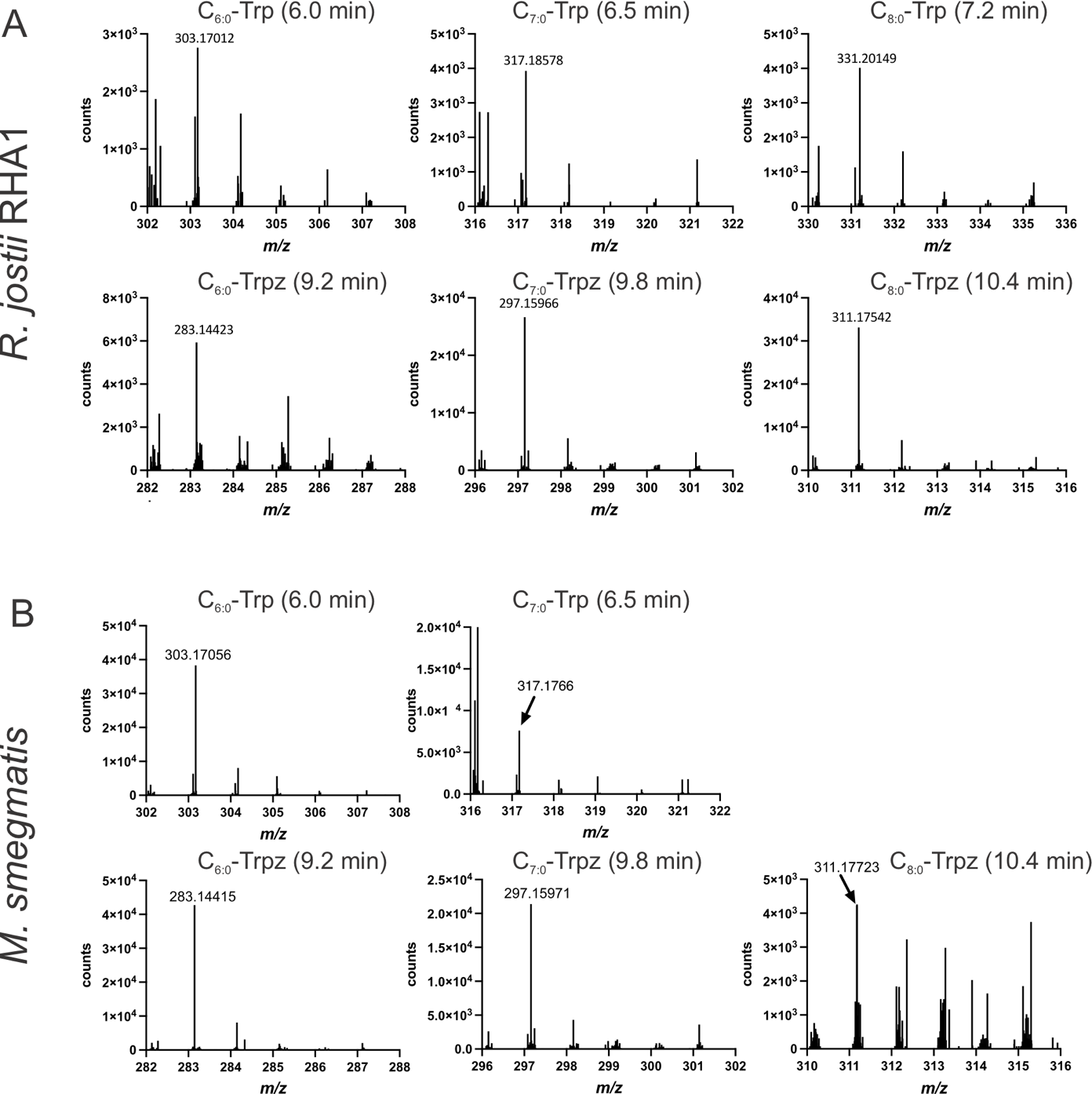


**Figure S****4. Mass spectra *N*-acyl tryptophan and tryptazolones in RHA1 and Msmeg.** The mass spectra of compounds for the expected *m/z* values for the [M+H]^+^ ion of C_6:0_-C_8:0_ tryptophan and tryptazolone detected in RHA1 (**A**) and Msmeg (**B**) are shown. Theoretical *m/z* values: C_6:0_-Trp, 303.17086; C_7:0_-Trp, 317.18652; C_8:0_-Trp, 331.20217; C_6:0_-Trpz, 283.14465; C_7:0_-Trpz, 297.16030; and C_8:0_-Trpz, 311.17595.


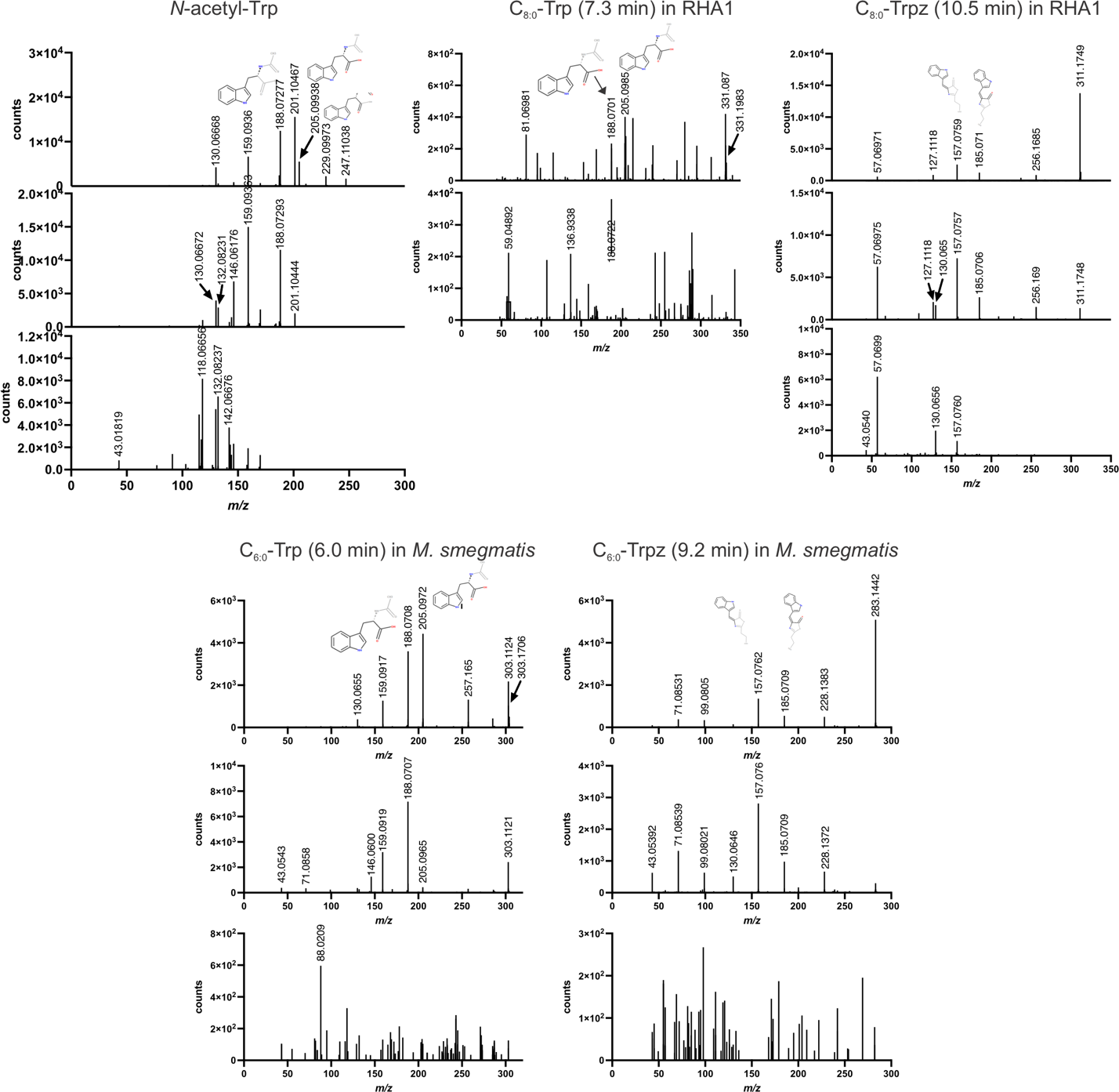


**Figure S****5. MS/MS fragmentation patterns of selected acylated tryptophan and tryptazolones from RHA1 and Msmeg.** Fragmentation of compounds with the expected *m/z* values for the [M+H]^+^ ion are displayed and compared to the pattern of commercially available *N*-acetyl tryptophan. Collision energies of 10, 20 and 40 V (top to bottom)


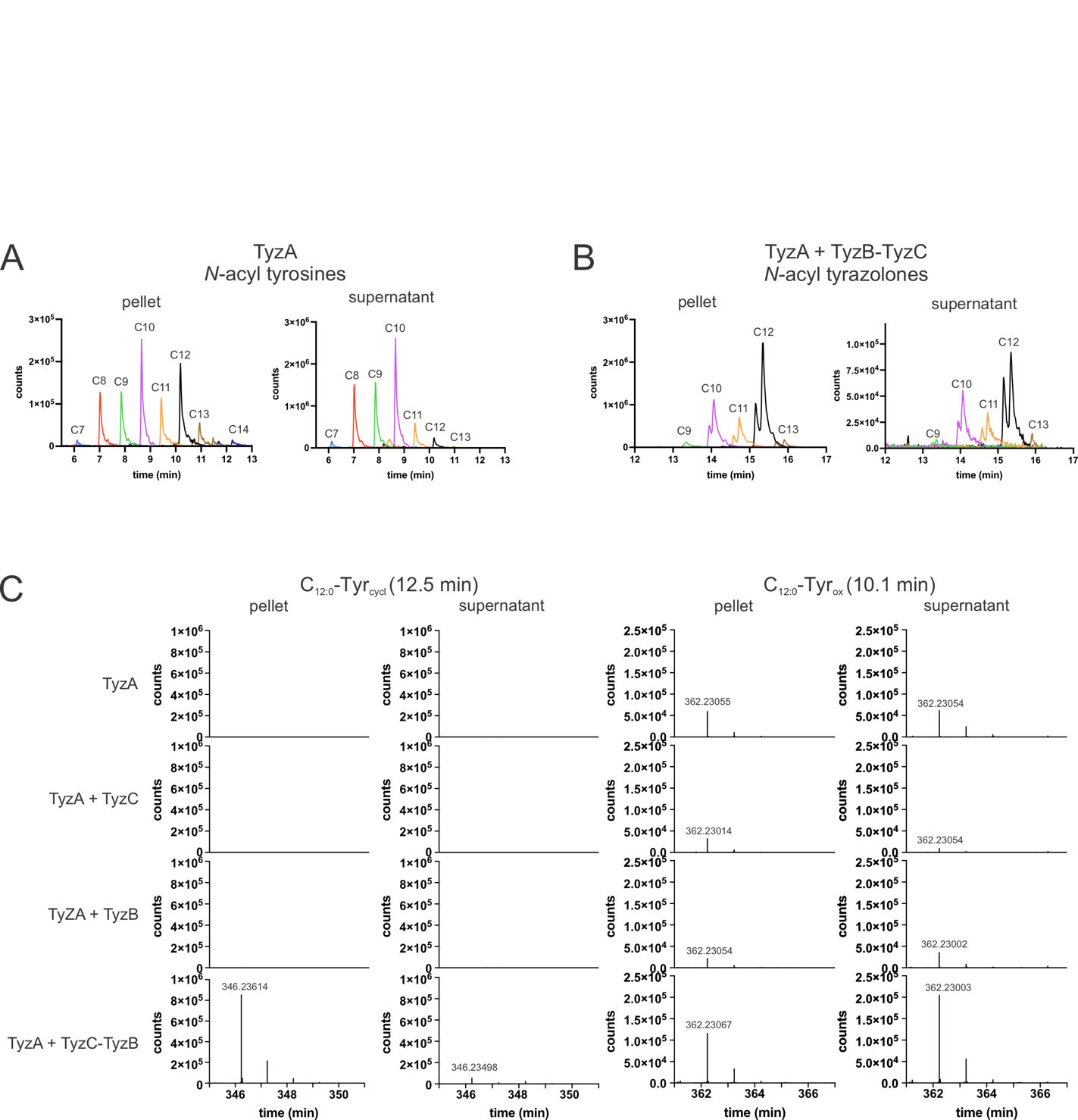


**Figure S****6. Acylated L-tyrosine and tyrazolones produced by TyzA-TyzC-TyzB from Mtb.** Using the RHA1 Δ*trzA*Δ*trzS* mutant as a host, strains were generated that produced either TyzA, TyzA and B, TyzA and C, or TyzA, B and C. Strains were grown in LB medium. Reaction products extracted from cell pellets and supernatants and were analyzed using LC-QTOF in positive mode. For simplicity, only data for products with fully saturated acyl chains are shown. EICs of strains producing TyzA (**A**) and TyzA, B and C (**B**) Mass spectra (**C**) for compounds corresponding to the expected *m/z* values for the [M-H]^+^ ion of acylated L-tyrosine and tyrazolones detected in the cell pellet and supernatant of strains are displayed. Detection of products is shown as detector counts against elution time. Theoretical *m/z* values: C_12:0_-Tyr_cycl_, 346.23821; and C_12:0_-Tyr_ox_, 362.23313.


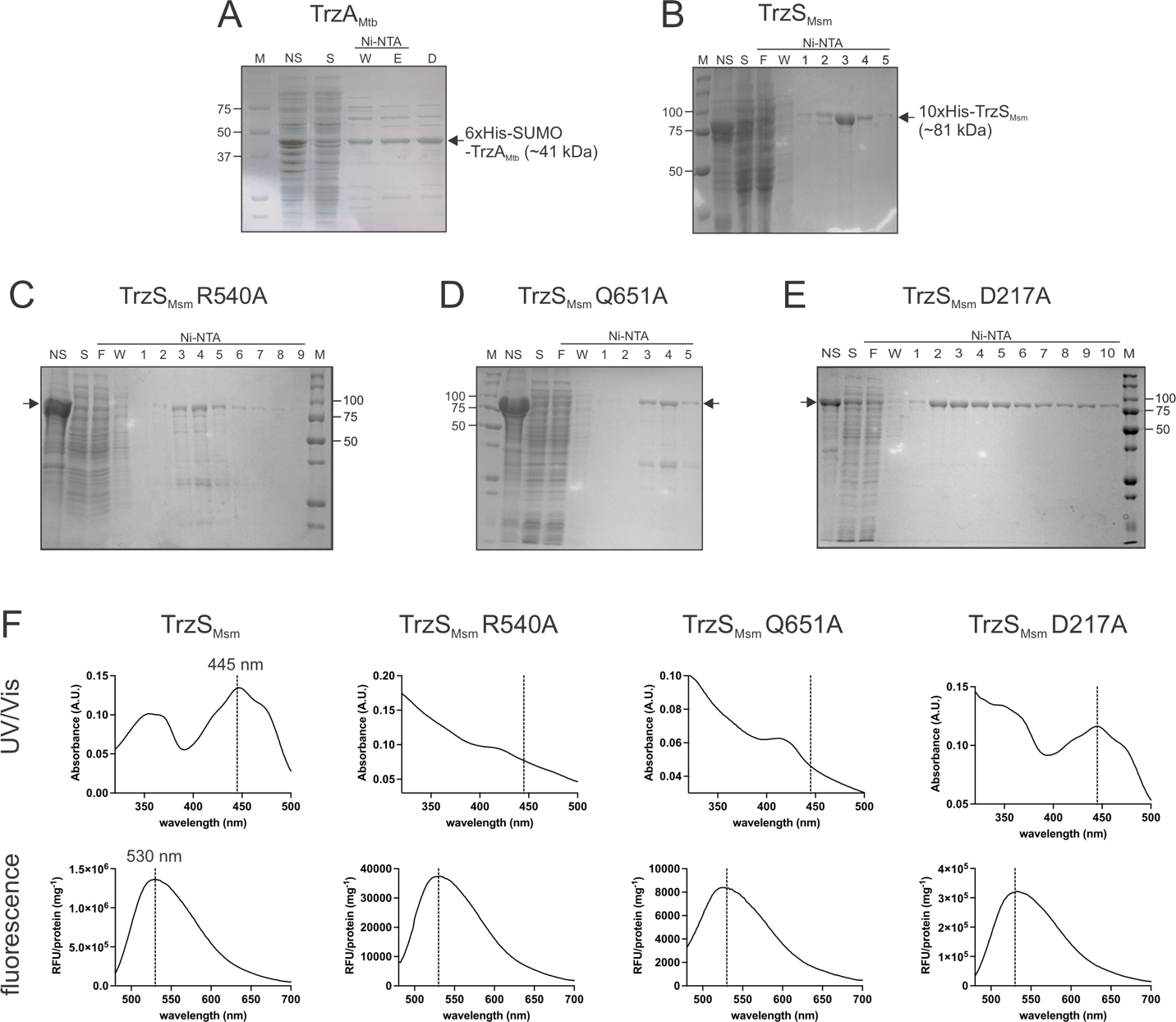


**Figure S****7. Purification of TrzA_Mtb_ and TrzS_Msmeg_. A**-**E**) N-terminally His-tagged TrzA_Mtb_ and TrzS_Msmeg_ were produced in *E. coli* and purified using Ni-NTA affinity chromatography. Eluted protein was dialyzed and the purified protein separated and visualized by Coomassie stained SDS-PAGE. The expected molecular weight of the His-tagged proteins is indicated by arrows. **F**) UV/Vis spectrum (top) and fluorescence emission spectrum (bottom, λ_ex_ = 440 nm) of wildtype and variant TrzS_Msmeg_. The FMN-specific absorbance peak at 445 nm and fluorescence emission peak at 530 nm are indicated by dashed lines. M, protein marker; NS, insoluble protein; S, soluble protein; W, wash fractions; E, elution fraction; D, dialyzed protein; Ni-NTA, samples eluted from Ni-NTA resin; 1-10, elution fractions; RFU, relative fluorescence units.


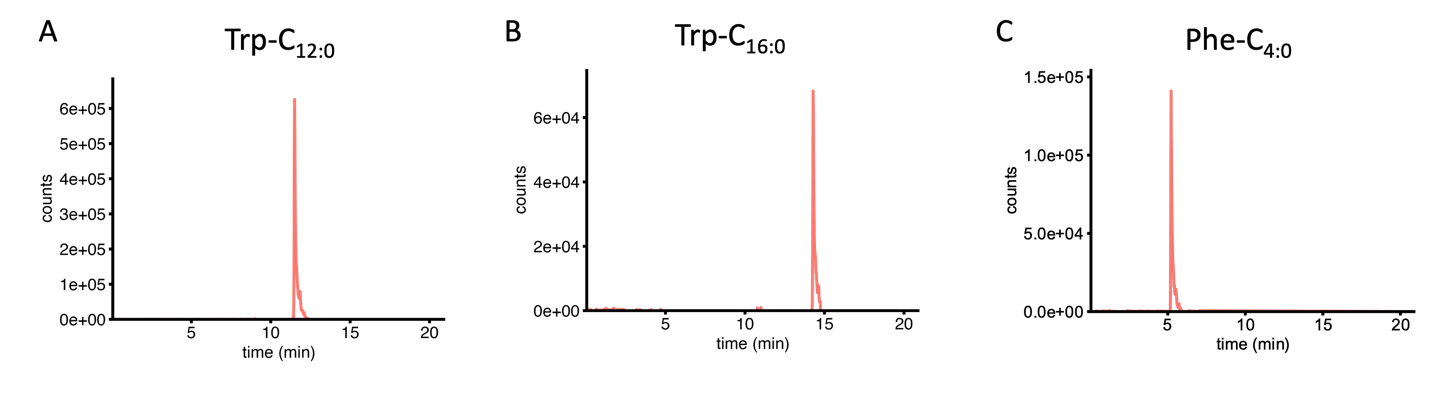


**Figure S****8. *In vitro* activity of TrzA_Mtb_.** Activity of purified enzyme was assessed using L-tryptophan or L-phenylalanine and C_12:0_-CoA **(A)**, C_16:0_-CoA **(B)** or C_4:0_-CoA **(C)**. Reactions contained 0.5 mM L-amino acid, 0.1 mM C_4:0_-CoA and 1 µM of TrzA_Mtb_ (20 mM Tris, 50 mM NaCl, pH 8.0) and were incubated at 21 °C for ~30 min. Reactions were quenched with acetonitrile and analyzed by LC-QTOF. EICs for *m/z* values for the [M+H]^+^ ion of expected products (detector counts vs. elution time).


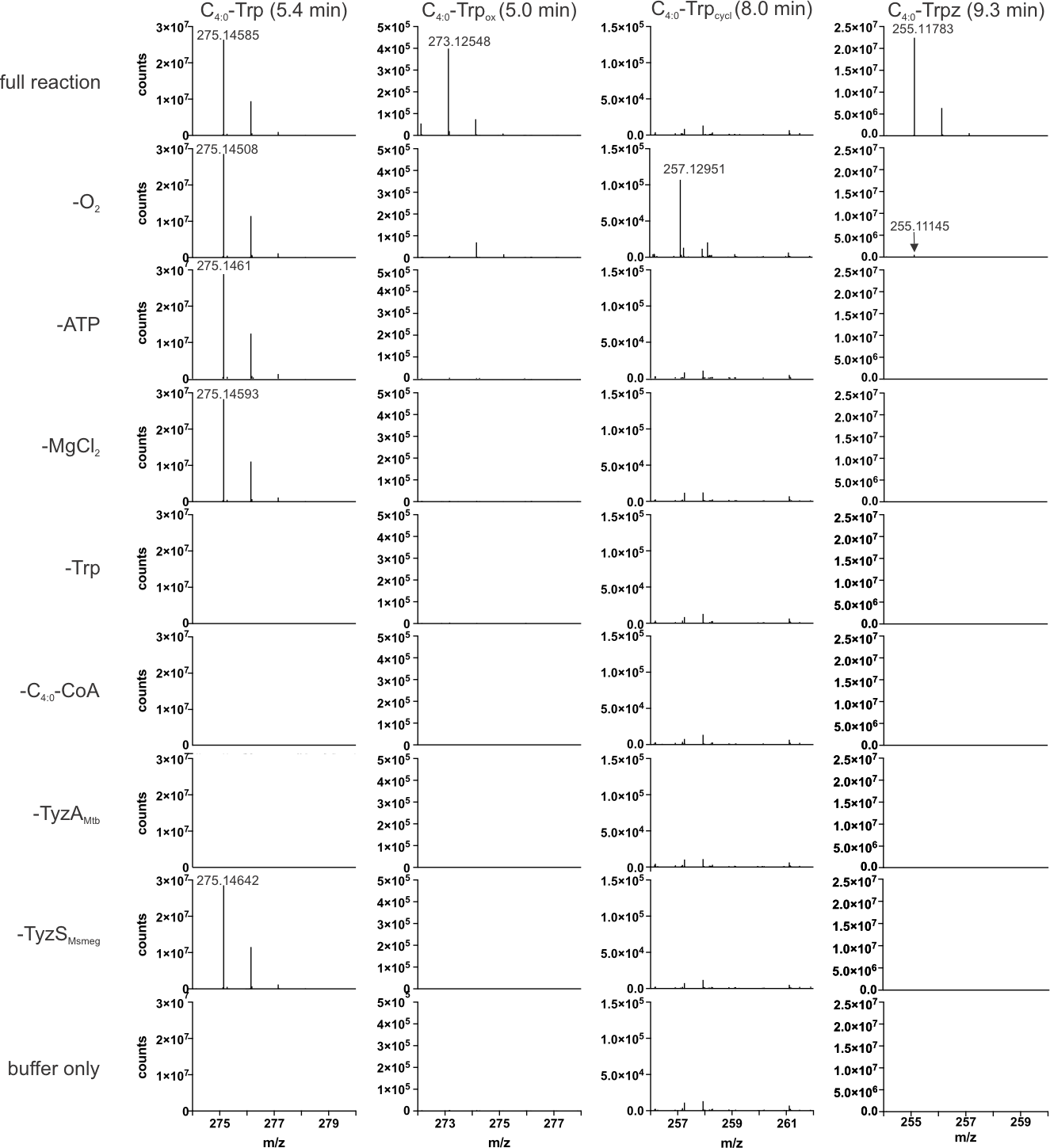


**Figure S****9. Mass spectra of C_4:0_-tryptazolone and produced intermediates from *in vitro* reactions.** Reactions contained 0.5 mM L-tryptophan, 0.1 mM C_4:0_-CoA, 0.5 mM ATP, 1 mM MgCl_2_, and 1 µM of TrzA_Mtb_ and TrzS_Msmeg_ and were incubated at 21 °C for approximately 30 minutes. Substrates were omitted from reactions as indicated to identify accumulating intermediates. Theoretical *m/z* values: C_4:0_-Trp, 275.13957; C_4:0_-Trp_ox_, 273.12392; C_4:0_-Trp_cycl_, 257.129; and C_4:0_-Trpz, 255.11335.


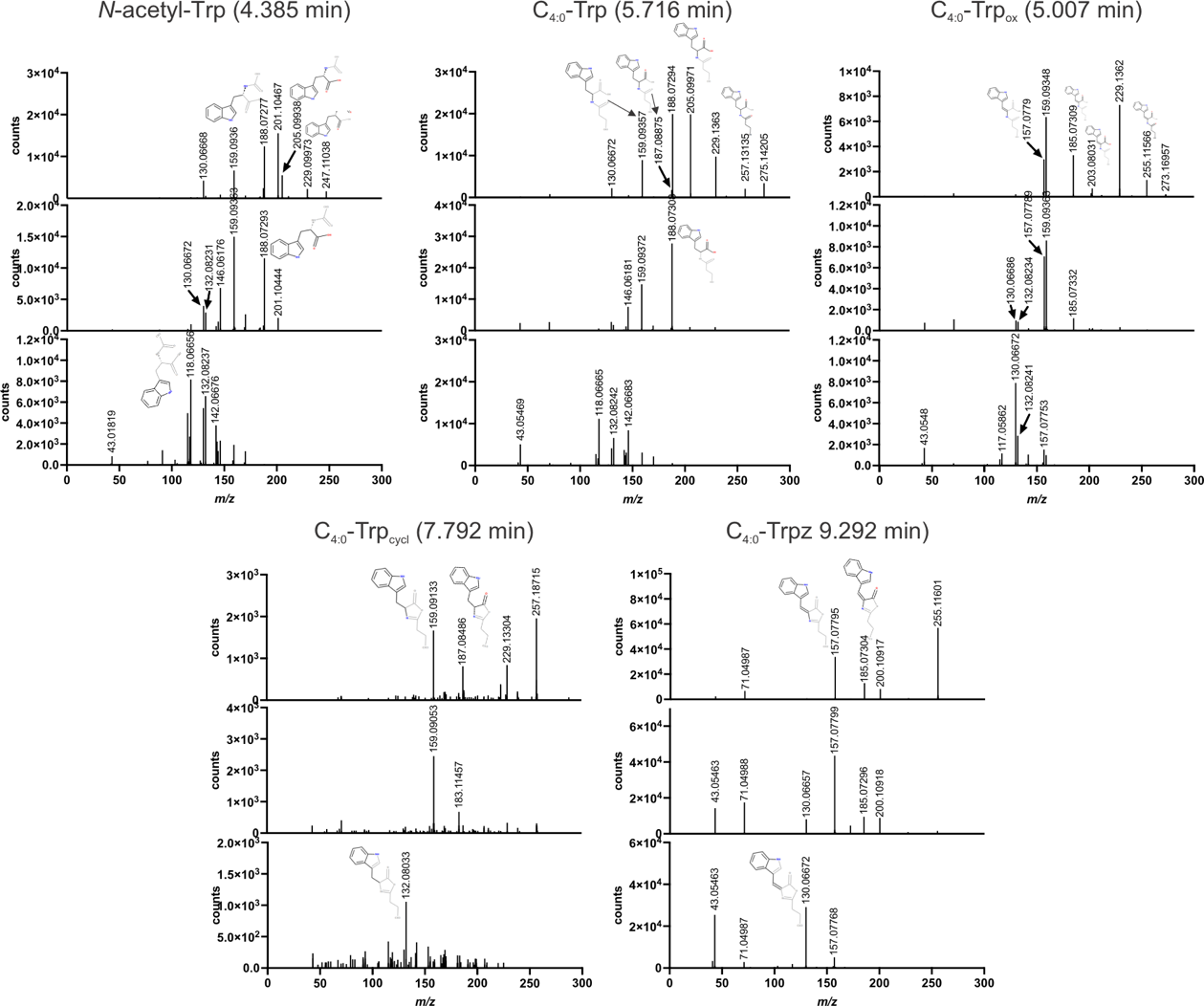


**Figure S****10. MS/MS fragmentation of TrzA_Mtb_ and TrzS_Msmeg_ reaction products.** An authentic standard, *N*-acetyl tryptophan was used and its fragmentation pattern compared to that of detected reaction products. In each series of panels, collision energies were 10, 20, and 40 V (top to bottom).


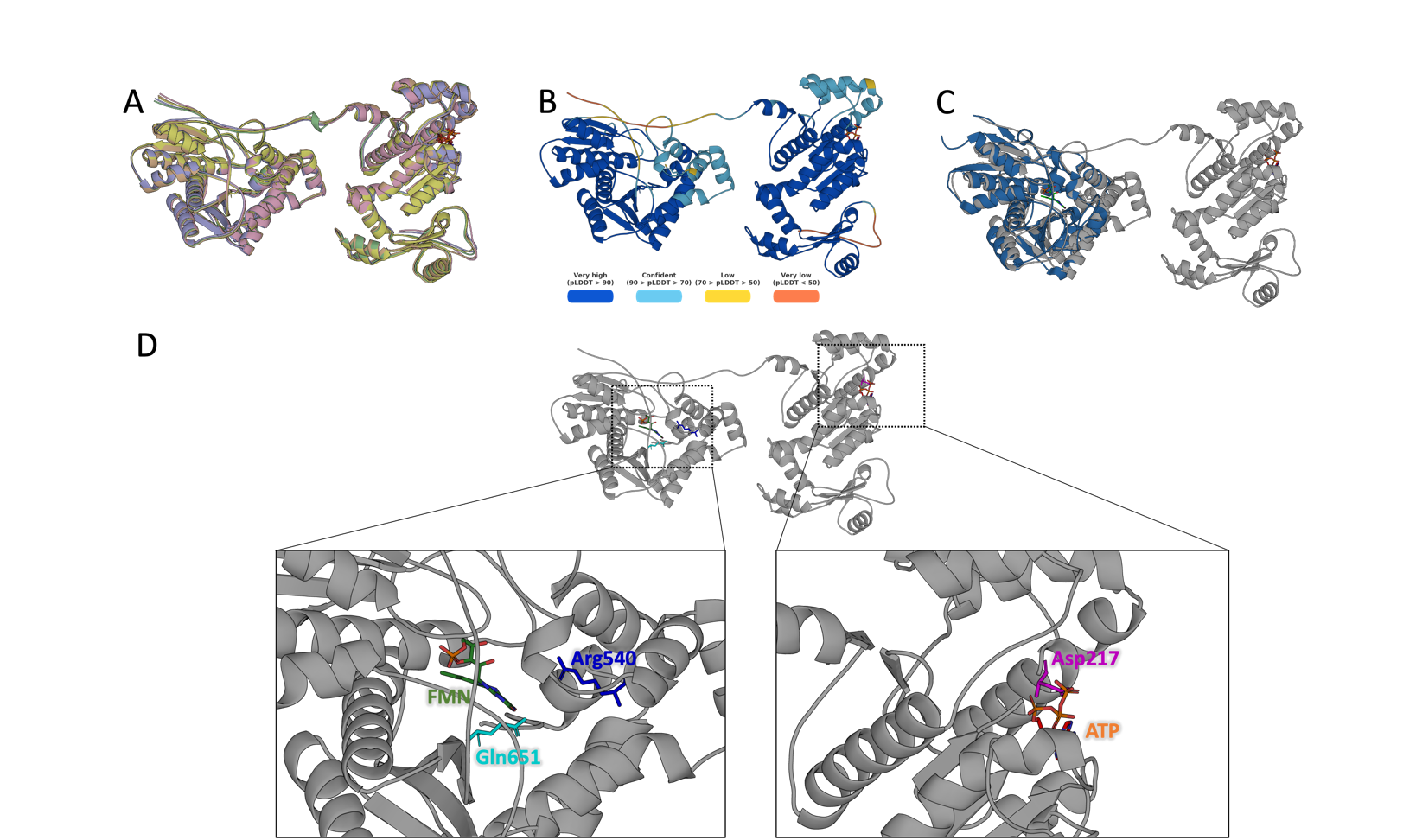


**Figure S****11. AlphaFold models of TrzS_Msmeg_.** The structure of the protein was predicted using AlphaFold 3 (8) as a monomer with ATP as a ligand. **A**) Overlay of the top 5 models. **B**) The highest ranked model of TrzS_Msmeg_ with residues colored according to the pIDDT score. **C**) Overlay of the highest ranked model of TrzS_Msmeg_ (grey) with the crystal structure of *M. smegmatis* Acg (Blue, PDB: 2YMV). **D**) Closeups of predicted active site residues that were substituted to disrupt function of the ThiF and FDO domains, respectively.


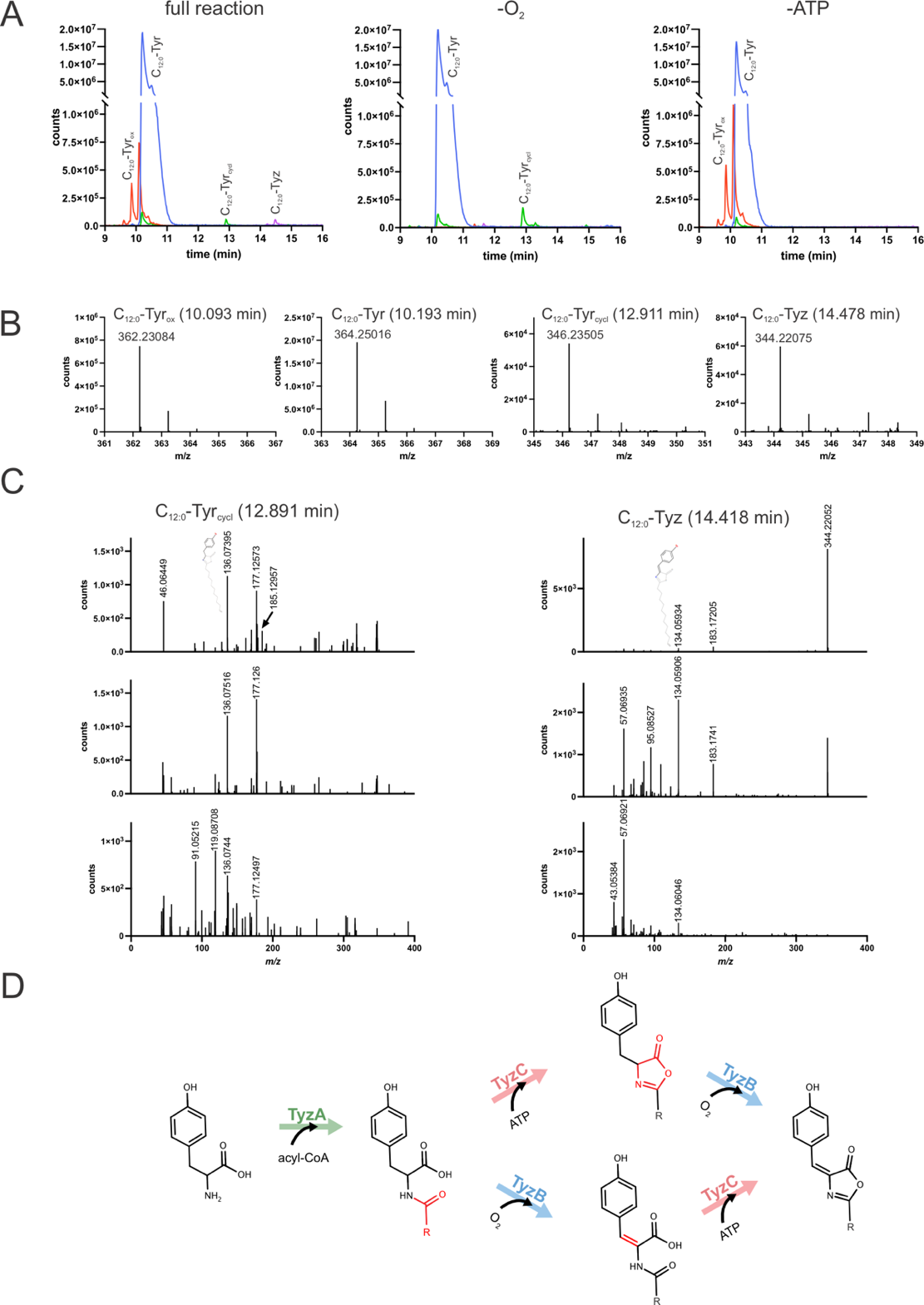


**Figure S****12. *In vitro* characterization of TyzA-TyzB-TyzC from Mtb.** Reactions contained 0.5 mM L-Tyr, 0.1 mM C_12:0_-CoA, 0.5 mM ATP, 1 mM MgCl_2_, and 1 µM of TyzA_Mtb_ and clarified lysate of RHA1 expressing *tyzC-tyzB*_Mtb_, and were incubated at 21 °C for ~30 min as described previously (4). Oxygen or ATP were omitted from the reaction to determine the accumulation of reaction intermediates. Since tryptazolones were susceptible to acids and alcohols, reactions were quenched with equal volumes of 100% acetonitrile, as compared to acetic acid in Grigg et al. (2023) (4). **A**) EICs of the expected *m/z* for the [M-H]^+^ ion of C12:0-tyrazolone and reaction intermediates. **B**/**C**) Mass spectra and MS/MS fragmentation patterns of the respective compounds at indicated retention times. **D**) Proposed reaction order of the Mtb TyzA-TyzC-TyzB system. Theoretical *m/z*: C_12:0_-Tyr_ox_: 362.23313, C_12:0_-Tyr: 364.24878, C_12:0_-Tyr_cycl_: 346.23821, C_12:0_-Tyz: 344.22257.


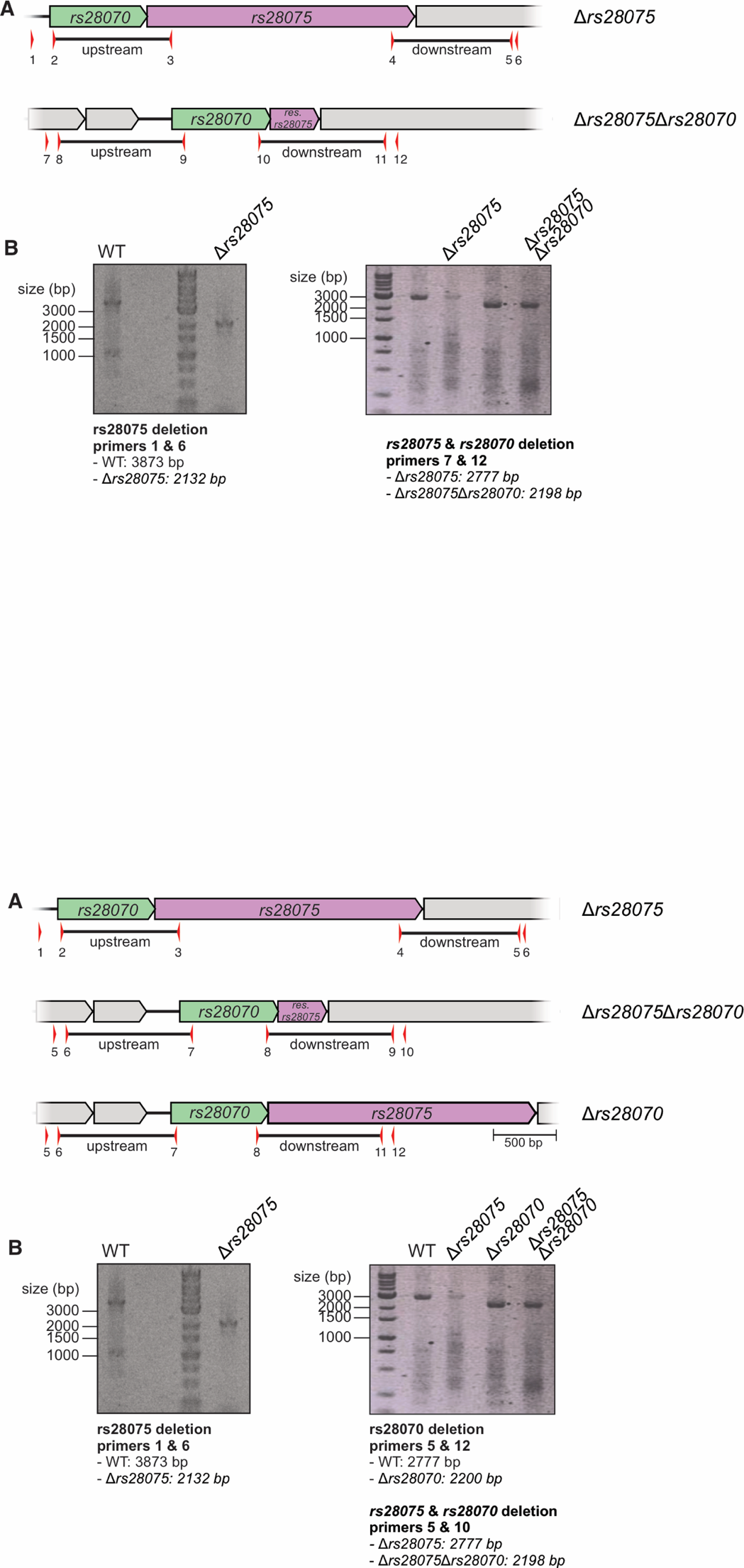


**Figure S****13. Construction of RHA1 deletion mutants. A**) Design of deletion constructs of *trzS* (*rs28075*) and *trzA* (*rs28070*) in RHA1. For each gene, the upstream flanking region contained the first 99 bp of the gene and 900 bp upstream of the start codon. Flanking regions were cloned using primers 2 and 3 for *trzS* and 8 and 9 for *trzA*. The downstream region contained the last 99 bp of the genes and an additional 900 bp downstream of the stop codon. Flanking regions were cloned using primers 4 and 5 for *trzS* and 10 and 11 for *trzA*. Primers indicated using numbered red arrows. Flanking regions indicated using black bars. **B**) Agarose gels of colony PCRs confirming the deletion of the respective genes.

# References

1. Studier, F. W., and Moffatt, B. A. (1986) Use of bacteriophage T7 RNA polymerase to direct selective high-level expression of cloned genes. *J. Mol. Biol.* **189**, 113–130

2. Simon, R., Priefer, U., and Pühler, A. (1983) A Broad Host Range Mobilization System for *In Vivo* Genetic Engineering: Transposon Mutagenesis in Gram Negative Bacteria. *Bio/Technology*. **1**, 784–791

3. Seto, M., Kimbara, K., Shimura, M., Hatta, T., Fukuda, M., and Yano, K. (1995) A Novel Transformation of Polychlorinated Biphenyls by *Rhodococcus* sp. Strain RHA1. *Appl. Environ. Microbiol.* **61**, 3353–3358

4. Grigg, J. C., Copp, J. N., Krekhno, J. M. C., Liu, J., Ibrahimova, A., and Eltis, L. D. (2023) Deciphering the biosynthesis of a novel lipid in *Mycobacterium tuberculosis* expands the known roles of the nitroreductase superfamily. *J. Biol. Chem.* 10.1016/j.jbc.2023.104924

5. Nakashima, N., and Tamura, T. (2004) Isolation and Characterization of a Rolling-Circle-Type Plasmid from *Rhodococcus erythropolis* and Application of the Plasmid to Multiple-Recombinant-Protein Expression. *Appl. Environ. Microbiol.* **70**, 5557–5568

6. Round, J. W., Robeck, L. D., and Eltis, L. D. (2021) An Integrative Toolbox for Synthetic Biology in *Rhodococcus*. *ACS Synth. Biol.* **10**, 2383–2395

7. van der Geize, R., Hessels, G. I., van Gerwen, R., van der Meijden, P., and Dijkhuizen, L. (2001) Unmarked gene deletion mutagenesis of *kstD*, encoding 3-ketosteroid Delta1-dehydrogenase, in *Rhodococcus erythropolis* SQ1 using *sacB* as counter-selectable marker. *FEMS Microbiol. Lett.* **205**, 197–202

8. Abramson, J., Adler, J., Dunger, J., Evans, R., Green, T., Pritzel, A., Ronneberger, O., Willmore, L., Ballard, A. J., Bambrick, J., Bodenstein, S. W., Evans, D. A., Hung, C.-C., O’Neill, M., Reiman, D., Tunyasuvunakool, K., Wu, Z., Žemgulytė, A., Arvaniti, E., Beattie, C., Bertolli, O., Bridgland, A., Cherepanov, A., Congreve, M., Cowen-Rivers, A. I., Cowie, A., Figurnov, M., Fuchs, F. B., Gladman, H., Jain, R., Khan, Y. A., Low, C. M. R., Perlin, K., Potapenko, A., Savy, P., Singh, S., Stecula, A., Thillaisundaram, A., Tong, C., Yakneen, S., Zhong, E. D., Zielinski, M., Žídek, A., Bapst, V., Kohli, P., Jaderberg, M., Hassabis, D., and Jumper, J. M. (2024) Accurate structure prediction of biomolecular interactions with AlphaFold 3. *Nature*. **630**, 493–500
